# Supplementary material for: A new class of capsid-targeting inhibitors that specifically block HIV-1 nuclear import
Source: EMBO Mol Med. 2024 Oct 2;16(11):13. doi: 10.1038/s44321-024-00143-w (PMC11555092; doi:10.1038/s44321-024-00143-w)
Supplement: Supplementary file 3 — Table EV3 [file 44321_2024_143_MOESM3_ESM.docx]

**Table EV3: Apparent rates of CA assembly and disassembly. (A)** Rate of HIV-1 CA assembly. Apparent rates of assembly were derived from the modified Hill expression: OD = OD_max_ . t^n^/(t_50_^n^ + t^n^), where t_50_ is the reaction half time, OD_max_ is the maximal signal and n is the Hill coefficient. The time constant and apparent association rate are given by **τ** = t_50_/ln(2) and *k_ass_* = ln(2)/t_50_ respectively. Errors are the standard deviation from at least three independent measurements. **(B)** Rate of HIV-1 disassembly. Apparent rates of disassembly were derived from the exponential decay function: OD = A.e^-t/τ^.where **τ** is the time constant and A is the decay amplitude. The reaction half time and apparent dissociation rate are given by t_50_ = ln(2).τ and *k_diss_* = 1/**τ** respectively. Errors are the standard deviation from at least three independent measurements.

**Table EV4A. Rate of HIV-1 CA assembly**

|  | **t_50_ (s)** | **n** | **OD_max_** | **τ (s)** | ***k_ass_* (s^-1^)** |
| --- | --- | --- | --- | --- | --- |
| **WT** | 144.1±0.9 | 2.1 | 2.03 | 207.9±1.3 | 0.0048±2.9e^-5^ |
| **H27** | 280.6±1.7 | 3.1 | 1.99 | 404.8±2.5 | 0.0025±1.52e^-5^ |
| **H84** | 343.1±1.4 | 3.5 | 1.69 | 495.0±2.0 | 0.0020±8.2e^-6^ |
| **H40** | 427.9±1.8 | 3.1 | 1.61 | 617.3±2.6 | 0.0016±6.8e^-6^ |
| **H70** | 391.6±1.5 | 3.1 | 1.64 | 565.0±2.1 | 0.0018±6.6e^-6^ |
| **Lenacapavir** | 54.3±0.8 | 1.5 | 2.21 | 78.4±1.2 | 0.0128±1.9e^-4^ |

Apparent rates of assembly were derived from the modified Hill expression: OD = OD_max_ . t^n^/(t_50_^n^ + t^n^), where t_50_ is the reaction half time, OD_max_ is the maximal signal and n is the Hill coefficient. The time constant and apparent association rate are given by **τ** = t_50_/ln(2) and *k_ass_* = ln(2)/t_50_ respectively. Errors are the standard deviation from at least three independent measurements.

**Table EV4B. Rate of HIV-1 disassembly**

|  | **τ (s)** | **A** | **t_50_ (s)** | ***k_diss_* (s^-1^)** |
| --- | --- | --- | --- | --- |
| **WT** | 62.1±0.25 | 1.71 | 43.1±0.17 | 0.016±6.4e^-5^ |
| **H27** | 73.3±0.15 | 1.24 | 50.8±0.10 | 0.014±2.7e^-5^ |
| **H84** | 56.5±0.24 | 1.49 | 39.2±0.16 | 0.018±7.4e^-5^ |
| **H40** | 100.6±0.10 | 1.53 | 69.7±0.007 | 0.010±1.0e^-5^ |
| **H70** | 111.1±0.53 | 1.38 | 77.0±0.37 | 0.009±4.3e-5 |
| **Lenacapavir** | n.m | / | / | / |

Apparent rates of disassembly were derived from the exponential decay function: OD = A.e^-t/τ^.where **τ** is the time constant and A is the decay amplitude. The reaction half time and apparent dissociation rate are given by t_50_ = ln(2).τ and *k_diss_* = 1/**τ** respectively. Errors are the standard deviation from at least three independent measurements.
