# Supplementary material for: A new class of capsid-targeting inhibitors that specifically block HIV-1 nuclear import
Source: EMBO Mol Med. 2024 Oct 2;16(11):13. doi: 10.1038/s44321-024-00143-w (PMC11555092; doi:10.1038/s44321-024-00143-w)
Supplement: Supplementary file 6 — Appendix [file 44321_2024_143_MOESM6_ESM.pdf]

Appendix

Appendix Figure S1.. 2
Appendix Figure S2.. 4
Appendix Figure S3.. 5
Appendix Figure S4.. 7
Appendix Figure S5.. 8
Appendix Figure S6.. 10
Appendix Figure S7.. 11
Appendix Figure S8.. 14
Appendix Figure S9.. 15
Appendix Figure S10.. 16
Appendix Figure S11.. 17
Appendix Table S1.. 19
- Fig. 2G.. 19
- Fig. 5D.. 20
- Fig. 6A.. 21
- Fig. 6B.. 23
- Fig. EV2.. 25

Appendix Figure S1: Chemical structures of the initial compounds assayed in **Fig. 1A** (compounds **1-40**).

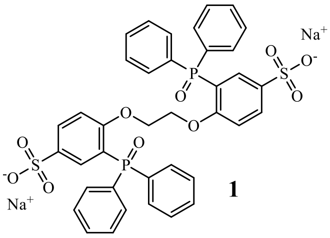

1

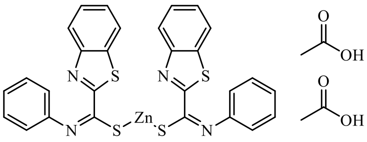

2

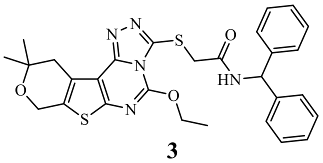

3

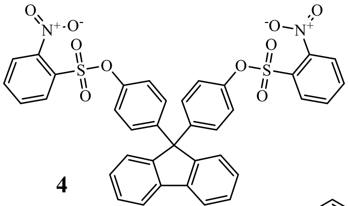

4

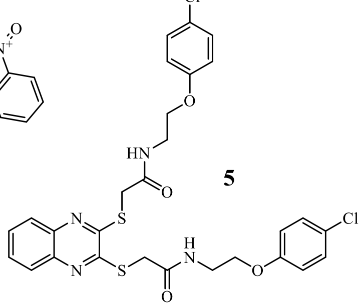

5

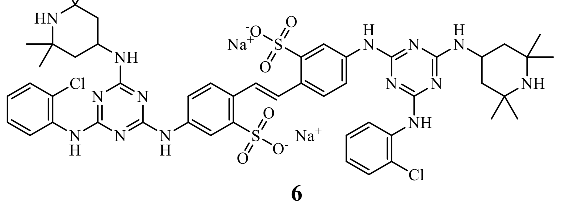

6

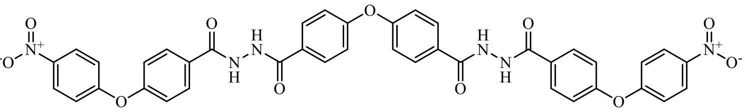

7

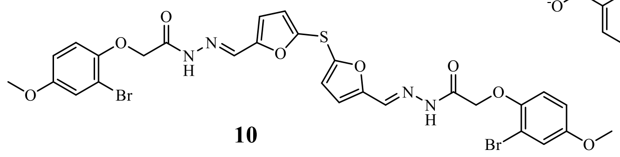

10

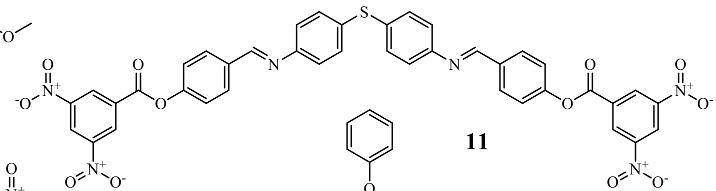

11

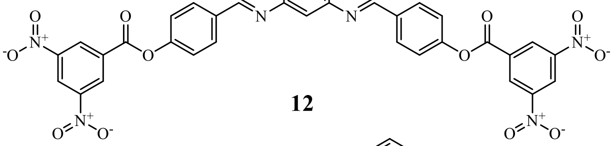

12

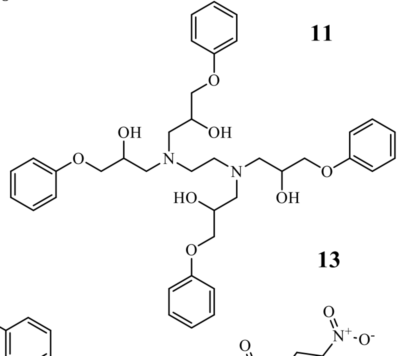

13

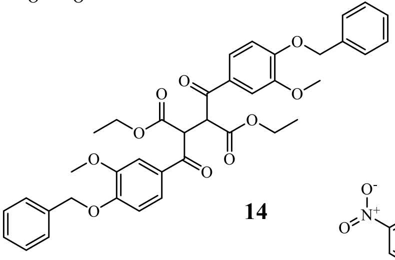

14

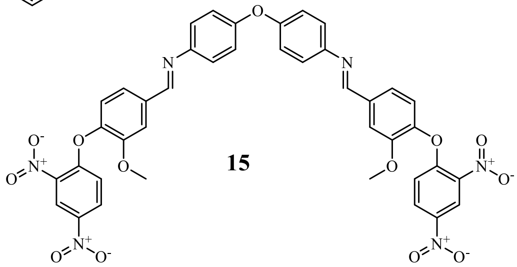

15

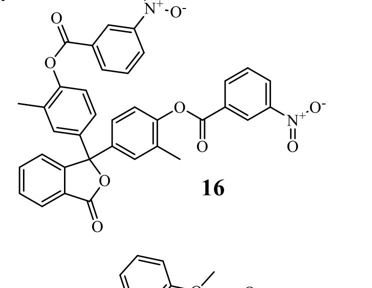

16

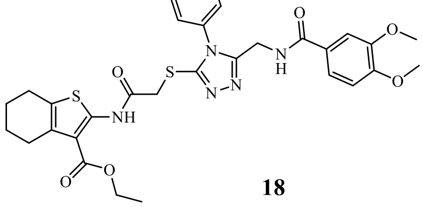

18

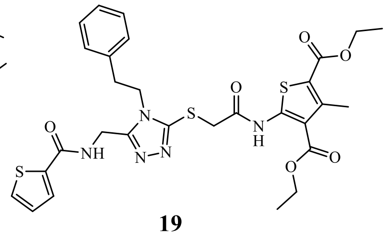

19

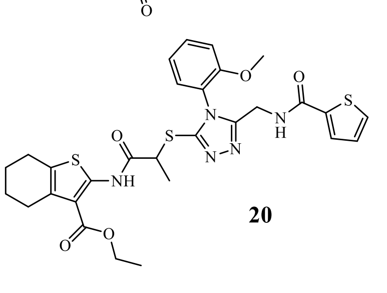

20

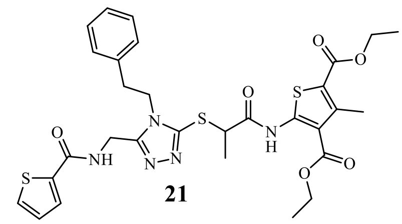

21

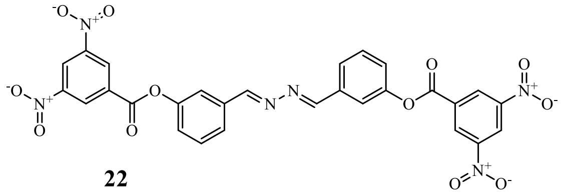

22

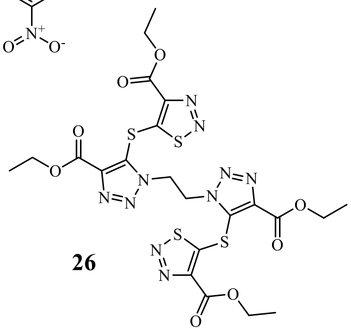

26

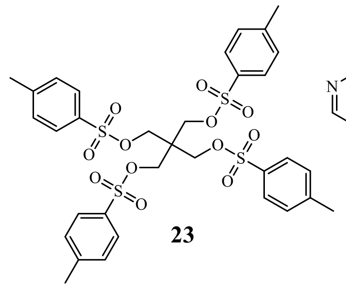

23

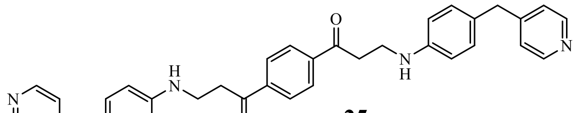

25

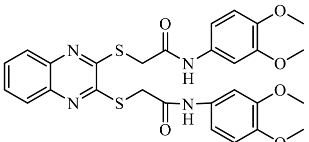

28

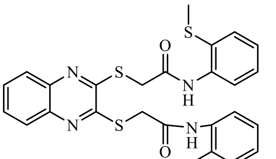

29

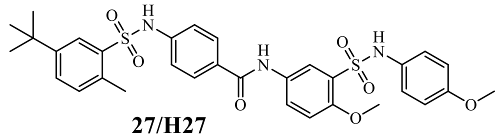

27/H27

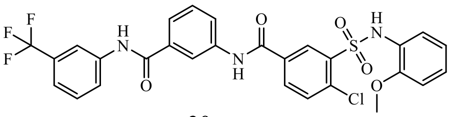

30

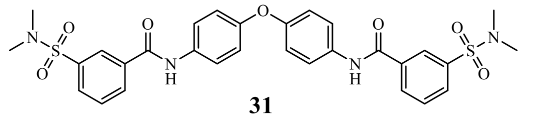

31

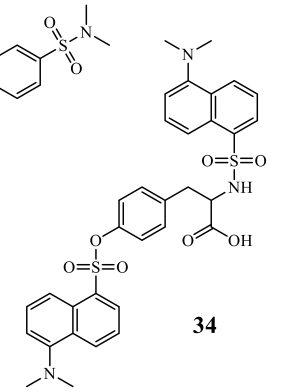

34

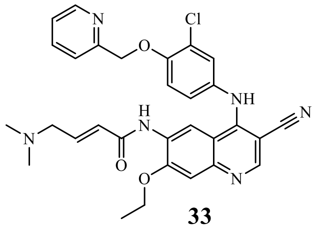

33

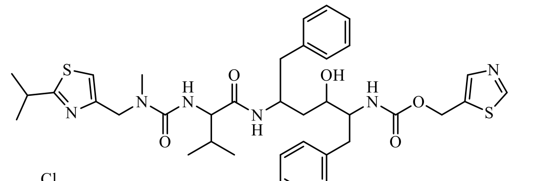

32

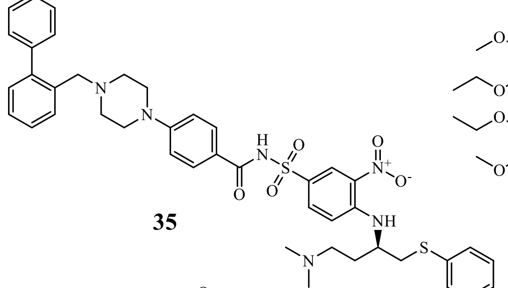

35

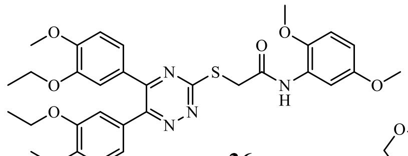

36

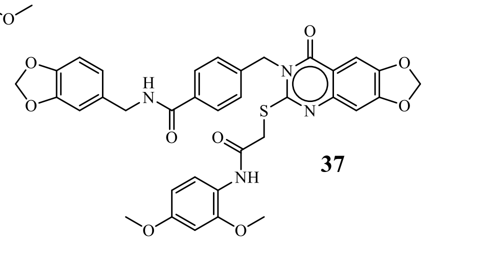

37

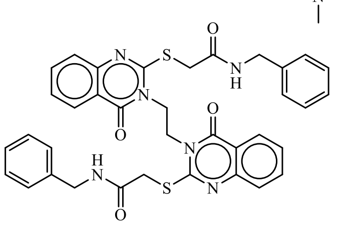

38

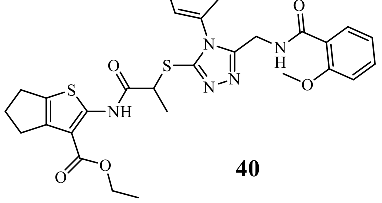

40

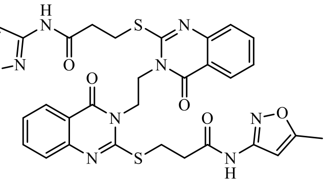

39

Appendix Figure S2: Binding affinity of H27 to HIV-1 hexamers. (A) SPR analysis of H27 (500 µM) binding to HIV-1 CA monomers (48,00RU), hexamers (14,000 RU, and to TRN-1 (14,000 RU) immobilized on a CM-5 sensor chip. **(B)** Determination of K_D of H27 for immobilized HIV-1 CA hexamers by one cycle kinetic titration at five increasing H27 concentrations (62, 125, 250, 500 and 1,000 µM).

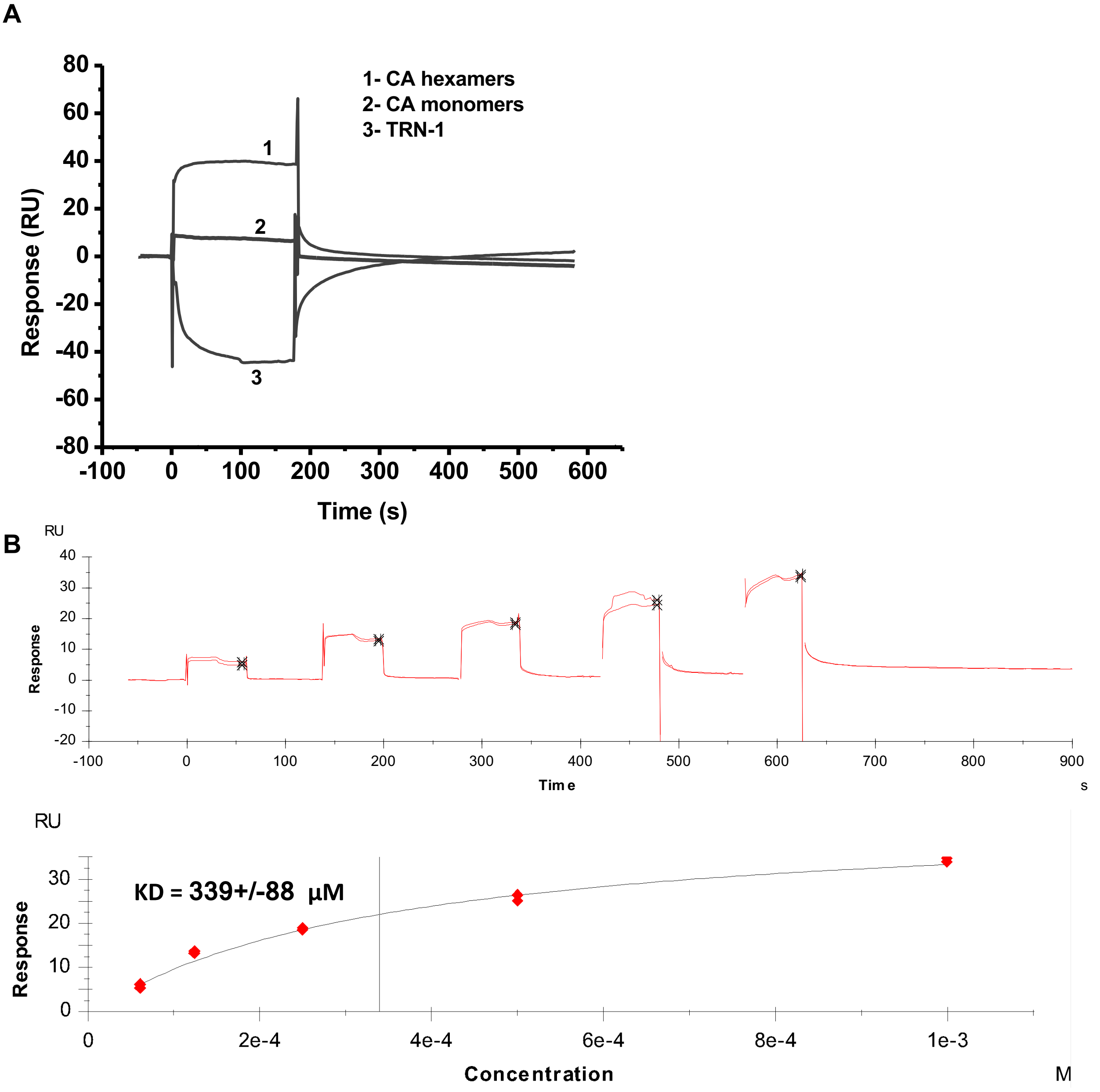

Appendix Figure S3: Effect of H27 on infection, cell viability and HIV-1 reverse transcription. (A) All tested HIV-1 were equally sensitive to H27, regardless of the tropism of their envelope. Infectivity in HeLa-R5 cells was assessed by ß-gal chemiluminescent assay at 48 hpi. Results show mean triplicate values ± SD from 2 independent experiments. For toxicity kinetics in **(B)** HeLa-R5 cells and **(C)** peripheral blood lymphocytes (PBL), cells were treated with H27 and compound **31** at concentrations ranging between 0.01 and 100 µM for 1, 3 or 6 days. Toxicity was assessed by Cell Titer assay. Results are representative of 2 independent experiments. Puromycin (2 µg/mL) was added to PBL as positive control. **(D)** Effect of H27 on reverse transcription at 7 hpi. HeLa-R5 cells were pre-treated with compounds at the indicated concentrations, then infected with VSV-G pseudotyped LAI, X4 tropic NL4-3 or R5-tropic BaL virus, at 1 ng p24/1,000 cells for 7 h. Compounds **31** and **40** were included as negative controls since they do not affect HIV-1 infection. Results are the mean of 2 independent experiments ± SEM normalised for the corresponding DMSO control. The reverse transcriptase inhibitor NVP (5 µM) were included as a negative control in all experiments. Reverse transcription (RT°) efficiency was assessed by qPCR amplification of late reverse transcripts (LRT) and normalised for Actin values.

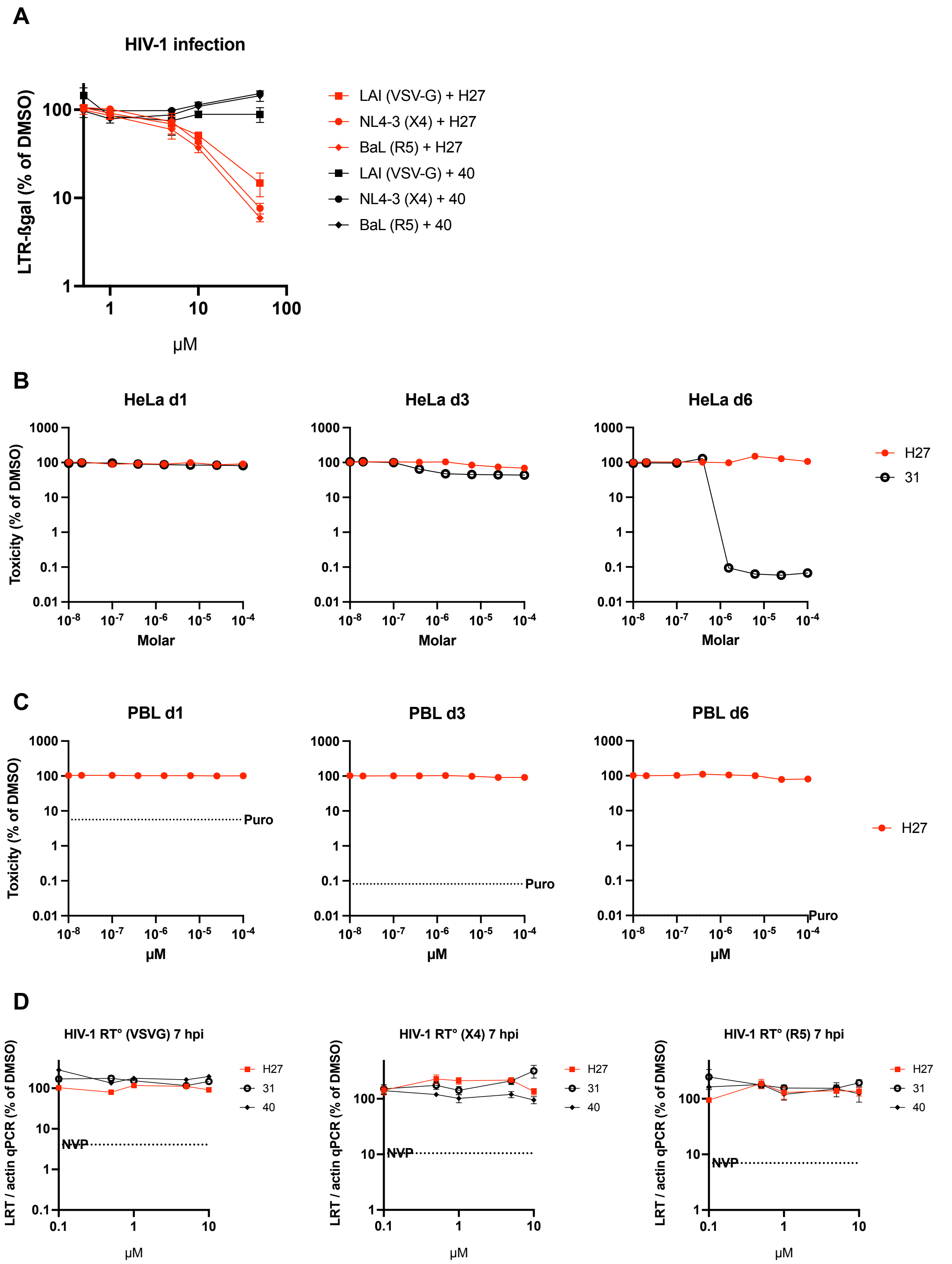
A
HIV-1 infection
LTR-ßgal (% of DMSO)
100
10
1
1
10
100
µM
LAI (VSV-G) + H27
NL4-3 (X4) + H27
BaL (R5) + H27
LAI (VSV-G) + 40
NL4-3 (X4) + 40
BaL (R5) + 40
B
HeLa d1
HeLa d3
HeLa d6
Toxicity (% of DMSO)
1000
100
10
1
0.1
0.01
10-8 10-7 10-6 10-5 10-4
Molar
H27
31
C
PBL d1
PBL d3
PBL d6
Toxicity (% of DMSO)
1000
100
10
1
0.1
0.01
10-8 10-7 10-6 10-5 10-4
µM
Puro
H27
D
HIV-1 RT° (VSVG) 7 hpi
HIV-1 RT° (X4) 7 hpi
HIV-1 RT° (R5) 7 hpi
LRT / actin qPCR (% of DMSO)
100
10
1
0.1
0.1
1
10
µM
NVP
H27
31
40

Appendix Figure S4: SPR measurement of AG3.0 antibody kinetics against monomeric and hexameric capsids. The affinity of AG3.0 antibody against monomeric and hexameric capsids were performed using one cycle kinetic titration by capture of AG3.0 (220 RU) on immobilized mouse anti-Fc and injecting monomeric CA from 10 to 160 nM and hexameric CA from 100 to 1600 nM, as two-fold dilution series. The K_D were calculated using a bivalent fitting model.

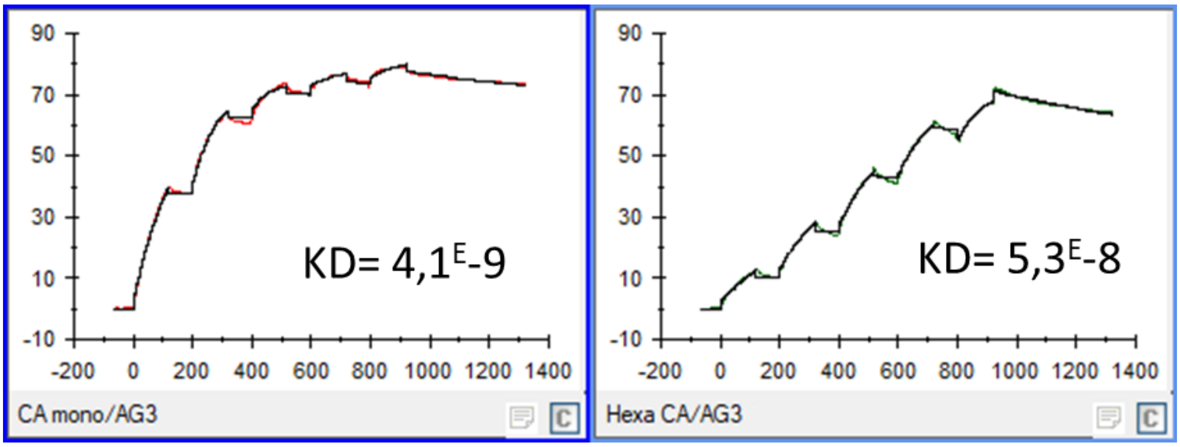

Sample	ka (1/Ms)	kd (1/s)	**KD(M)**	Chi² (RU²)
CA mono	1,69E+05	6,96E-04	**4,12E-09**	0,426
Hexa CA	5,93E+03	3,20E-04	**5,39E-08**	0,478

Appendix Figure S5: CA localisation following treatment with H27, PF74 or LEN. HeLa-R5 were treated with H27 (3 µM), PF74 (1 µM), LEN (9 nM), or DMSO (0.5%), then inoculated with LAI-VSV-G at 1 ng p24/1,000 cells in serum-free medium to promote cell attachment, then incubated in 10% FCS for a further 2 h or 6 h to allow infection to proceed. Samples were fixed, permeabilised and labelled with anti-CA and -Nup214 antibodies, and stained with Hoechst. Images were acquired on a LSM880 Airyscan confocal microscope with a 63 × objective. The cell periphery was delineated using the line tool from FiJi using cell autofluorescence. Arrows point to intranuclear CA spots. Images are representative of 3 independent experiments.

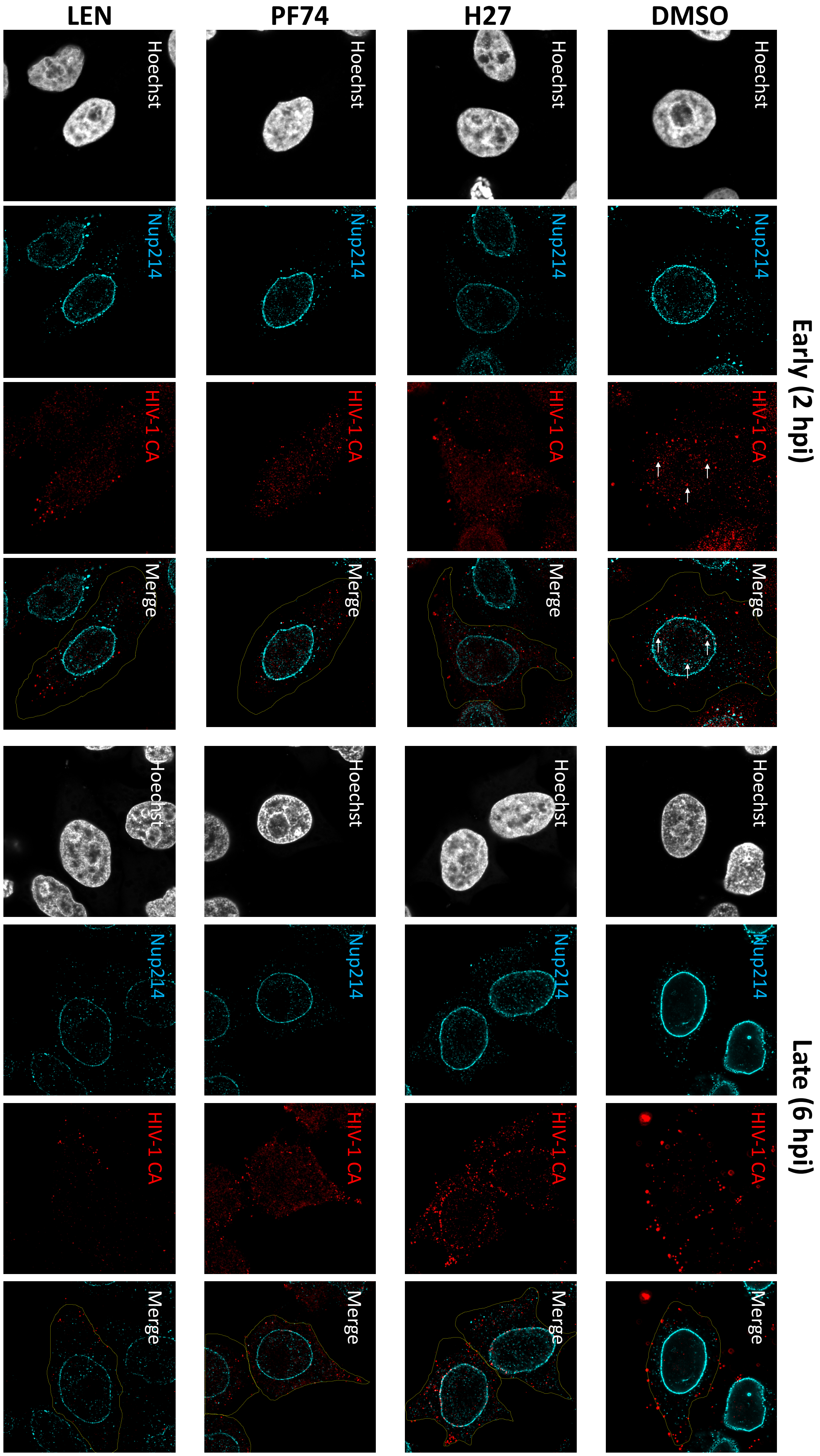
LEN
PF74
H27
DMSO
Hoechst
Nup214
HIV-1 CA
Merge
Early (2 hpi)
Late (6 hpi)

Appendix Figure S6: Effect of H27 on the subcellular localization of shuttling proteins. (A) HeLa-R5 cells were treated with DMSO or H27 (10 µM) for 8 h, then fixed and labelled with antibodies recognizing the shuttling proteins TRN-1, CPSF6 and PML. **(B)** HeLa-R5 cells were pre-treated with DMSO or H27 (10 µM) for 2 h, then stimulated with Influenza A virus (WSN strain) at 0.1 TCID50/cell. Cells were fixed after 6 h and labelled with antibodies recognizing the innate immune transcription factors IRF-3 and NF-κB or the viral nucleoprotein (NP). Scale bars = 10 µm.

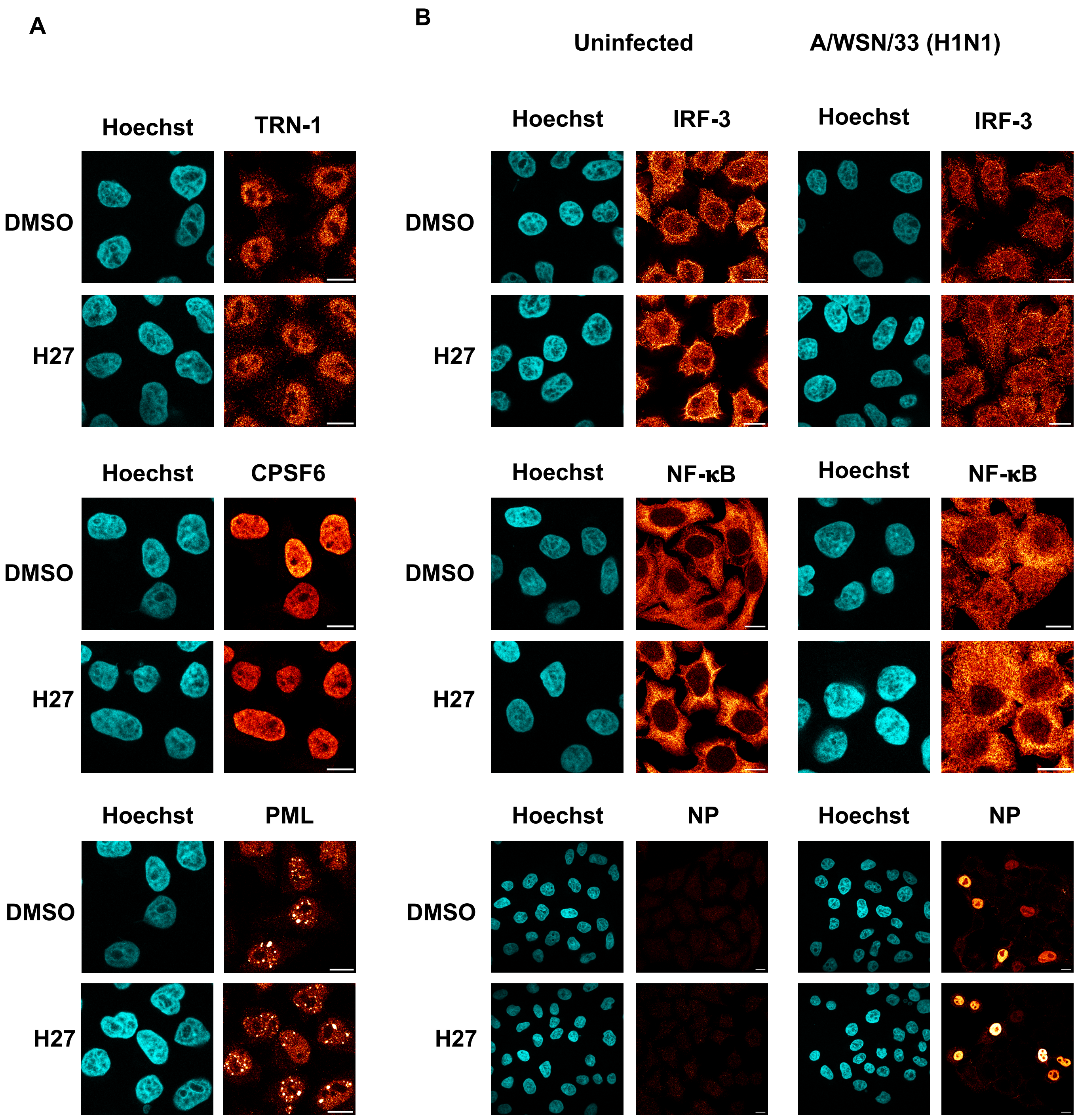

Appendix Figure S7: Infectivity and toxicity of H27 and analogues 41 - 85. The infectivity and toxicity of H27 and its structural analogues (compounds **41** to **85**) were assessed in HeLa-R5 cells. Infectivity was determined at 48 hpi following infection with HIV-1-VSV-G at 0.1 ng p24/1,000 cells, and toxicity was assessed in separate plates by Cell Titer assay in HeLa-R5 cells. Results show the mean ± SD of 2 independent experiments performed in triplicate.

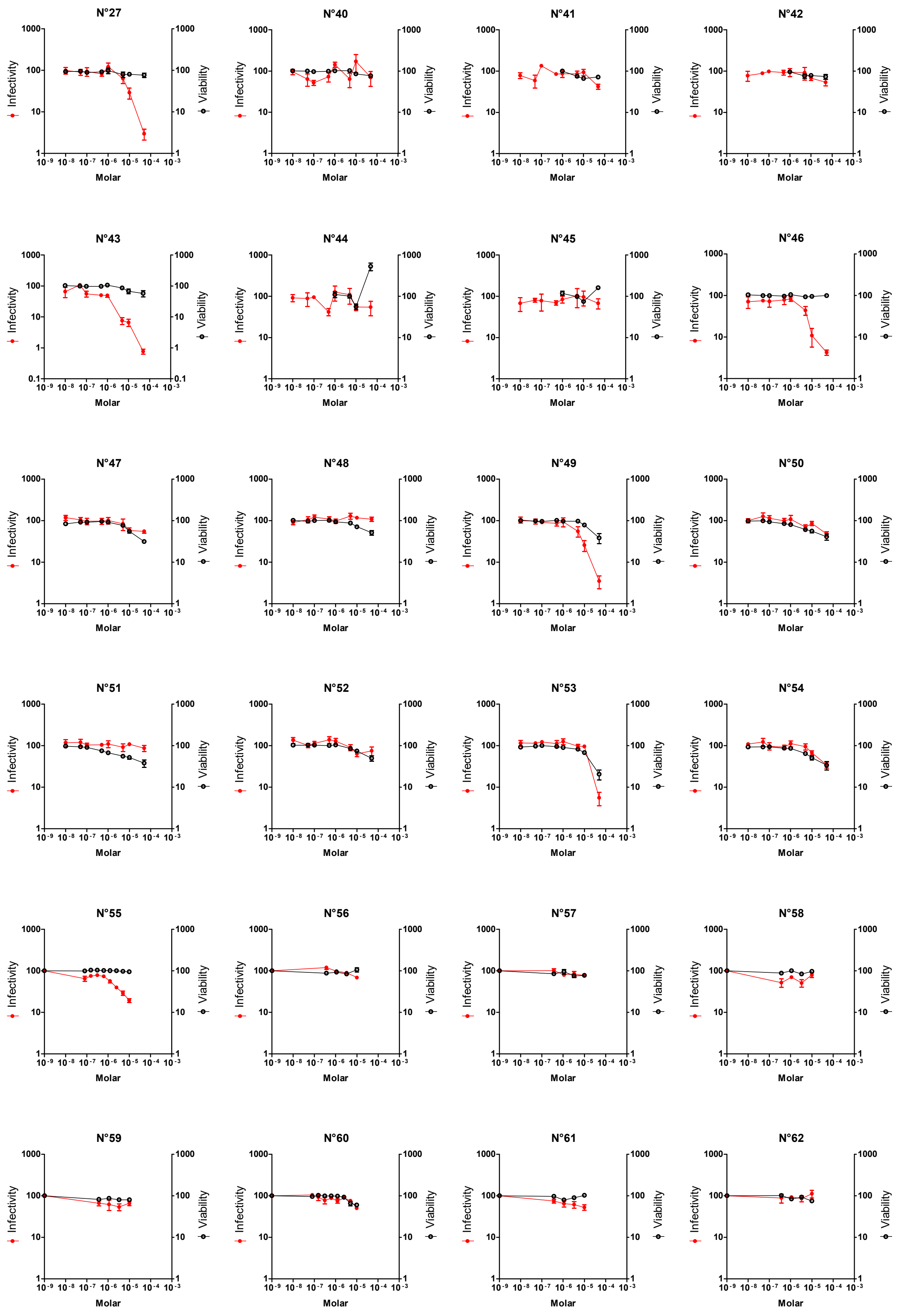
N°27
N°40
N°41
N°42
N°43
N°44
N°45
N°46
N°47
N°48
N°49
N°50
N°51
N°52
N°53
N°54
N°55
N°56
N°57
N°58
N°59
N°60
N°61
N°62
Infectivity
Viability
Molar

N°63

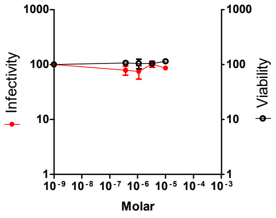

N°64

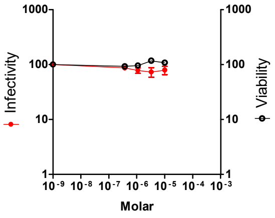

N°65

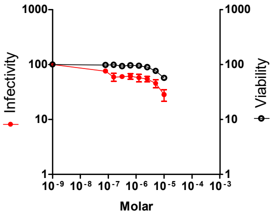

N°66

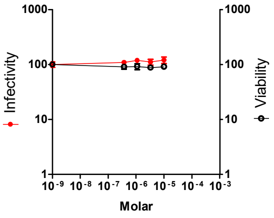

N°67

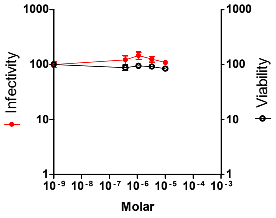

N°68

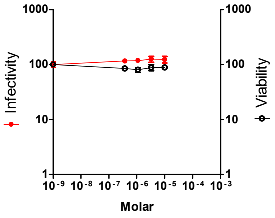

N°69

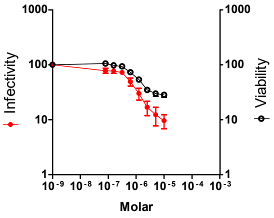

N°70

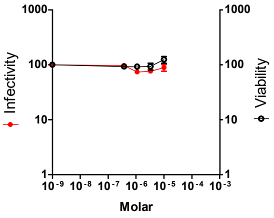

N°71

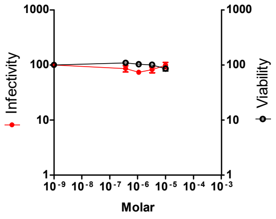

N°72

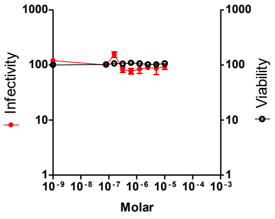

N°73

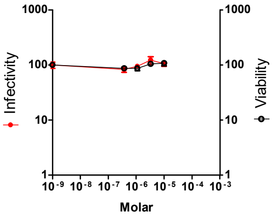

N°74

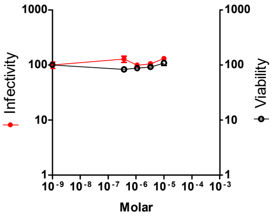

N°75

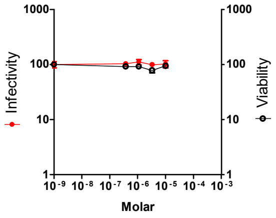

N°76

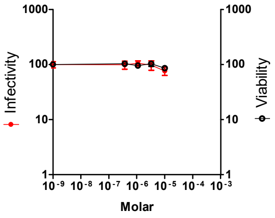

N°77

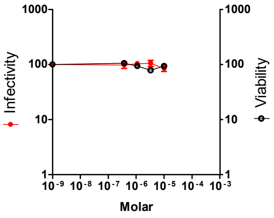

N°78

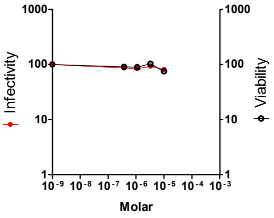

N°79

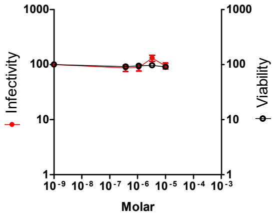

N°80

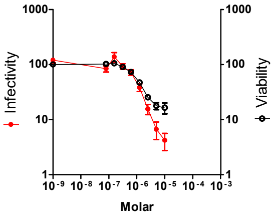

N°81

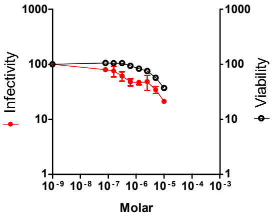

N°82

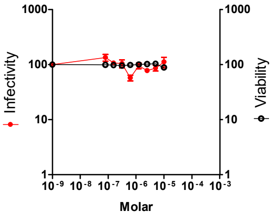

N°83

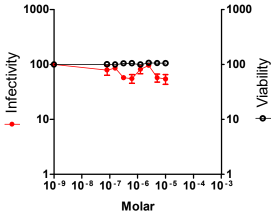

N°84

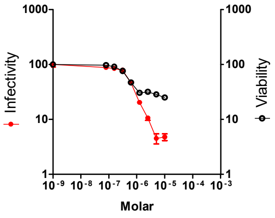

N°85

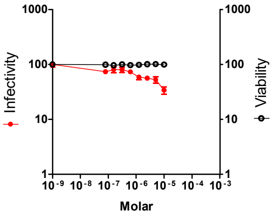

Appendix Figure S8: Sequence alignments of primate lentiviral CA. Alignment of the capsid of HIV-1M and SIVgsn-99CM71, SIVmon-99CMCML1, SIVagmSAB, SIVmac251, HIV-2_{ROD}, SIVmnd2 and SIVcolCGU1. Dots represent fully conserved residues and deletions are indicated with dashes. The PY-NLS recognised by TRN-1 is highlighted in yellow.

```
                      10        20        30        40        50        60        70        80        90
                       |         |         |         |         |         |         |         |         |
HIV-1M        PIVQNLQGQMVHQAISPRTLNAWVKVIEEKAFSPEVIPMFSALSEGATPQDLNTMLNTVGGHQAAMQMLKETINEEAAEWDRLHPVHAGPIAPGQ
SIVgsn        ...R.A...FQ...LNS.V.KT..SIV...K.A..TVAL.Q..T..CI.Y.M.Q...A..DY.G.V.II.DV...Q.....L...QP.A.QPVAG
SIVmon        .V.RTQG.GFQ...VE..L.KT..Q.....K.A...VAL.Q.....MI.Y.I.QL..AI.D..G.L.II.DV......N..LV..QPPQ.QPNAG
SIVagmSAB     .-IVSVNN.W...PL...............K..A..V......A...I.Y.I.Q...A..E..G.L.IV.DV......D..LR..PPQQ.P.Q.V
SIVmac251     .-..QIG.NY..LPL..........L....K.GA..V.G.Q.....C..Y.I.Q...C..D......IIRDI......D..LQ..QP.-.Q-Q..
HIV2ROD       .-..HVG.NYT.IPL..........LV...K.GA..V.G.Q.....C..Y.I.Q...C..D......IIR.I.........VQ..IPG-.LPA..
SIVmnd2       .-I.VINQTP...G...........C....K....IV...I.....CI.Y...G...AI.D..G.L.IV.DV......D..LR..PVG-.MPQ.V
SIVcol        ...TGP..-P...PL.....G....CV.G-GIA.SLA...L.Y.T..IAY.M.L...ILDT..GFP.V..DE..KK.E.Y.L....QQ-.QQQ.A

                     110       120       130       140       150       160       170       180       190
                       |         |         |         |         |         |         |         |         |
HIV-1M        GSDIAGTTSTLQEQIGWMTN-NPPIPVGEIYKRWIILGLNKIVRMYSPVSILDIRQGPKEPFRDYVDRFYKTLRAEQATQEVKNWMTETLLVQNA
SIVgsn        .A....V...PN...E.I.RQ.N.VN.AD..RK......QRC.K..N..N..G.K.......KE.....F.C......D.A......QL......
SIVmon        .A....VS..P.Q..E.I.RA.N.VQ.SD..RK.V....QRC.K..N..N....K.......K........C.....TD.A......Q.......
SIVagmSAB     ..........I....E.T.RAQNAVN..N...G......Q.C.K..N..N....K.......K........A.....TDPA......QS..I...
SIVmac251     .........SVD...Q..YRQQN.....N..R...Q...Q.C....N.TN...VK.......QS.......S.....TDAA......Q...I...
HIV2ROD       ..........VE...Q..FRPQN.V...N..R...QI..Q.C....N.TN....K.......QS.......S.....TDPA......Q.......
SIVmnd2       .N.......SIE...E.T.RQQDQVN..G...Q..V...Q.C.S..N..N....K.......K........A....RTDPQ..T...Q...I...
SIVcol        A...T.N..SVA..VA.GEP------IAN..RG..VQS.E.VIQIAR.S.V......S..D.KS......SA....P.AG.I.A..ANN..I.H.

                     210       220       230
                       |         |         |
HIV-1M        TILKALGPAATLEEMMTACQGVGGPGHKARV
SIVgsn        L....MPG-.S....L.........T..S.L
SIVmon        L....MPG-.S..D.LQ........M..S.I
SIVagmSAB     .V..G..MNP.....L.....I..AQ....L
SIVmac251     LV..G..VNP.....L..........Q...L
HIV2ROD       LV..G..MNP.....L..........Q...L
SIVmnd2       AT..G..MNP.....LL........KY..QM
SIVcol        RI..G.QK-PS..D.LA........D...K.
```

Appendix Figure S9: Listing of CA residues from two adjacent monomers within a hexamer that contact H27. Residues either from **(A)** HIV-1 or from **(B)** SIVmac in contact with H27 (ranked by contact frequency during MD simulations showing only high frequency contact (> 10%)). Sustained binding observed with residues belonging to the PR loop are highlighted in yellow and common residues between HIV-1 and SIVmac CA are underlined in grey. **(C)** PY-NLS that is recognized by TRN-1, and corresponding residues for SIVmac251. Residues implicated in sustained contact with H27 are highlighted in yellow.

A

Ligand	ResName	Monomer	ResNumber	Contact (%)
H27	**PRO**	A	125	59.3
H27	**PRO**	A	122	57.3
H27	**MET**	A	96	52
H27	**PRO**	A	123	51.1
H27	**GLN**	B	112	46.4
H27	**GLN**	A	4	45.8
H27	**THR**	B	110	44.6
H27	**ILE**	B	115	44.3
H27	**ILE**	A	124	41.9
H27	**GLY**	B	116	40.6
H27	**ILE**	A	129	39.7
H27	**TRP**	A	80	39.4
H27	**PRO**	B	93	37.3
H27	**ARG**	A	132	36.1
H27	**THR**	B	119	36
H27	**GLY**	B	94	35.9
H27	**GLN**	B	95	34.8
H27	**LEU**	A	6	34.1
H27	**HIS**	B	12	33.2
H27	**GLU**	B	113	31.7
H27	**HIS**	A	84	30.4
H27	**VAL**	B	11	30.1
H27	**MET**	A	10	29.6
H27	**HIS**	A	120	29.1
H27	**PRO**	A	99	28.5
H27	**ASN**	A	5	28
H27	**HIS**	B	120	27.9
H27	**MET**	B	10	25.2
H27	**LEU**	A	83	24.7
H27	**TRP**	B	117	24.2
H27	**ALA**	B	92	23.3
H27	**GLN**	B	9	20.8
H27	**GLU**	A	98	20.5
H27	**GLU**	A	128	19.6
H27	**MET**	A	118	18.7
H27	**GLU**	A	79	16.7
H27	**ILE**	B	124	16.4
H27	**TRP**	A	133	15.7
H27	**ASN**	A	121	15.4
H27	**ARG**	B	97	13.7
H27	**GLN**	A	9	13
H27	**ILE**	A	2	10

B

Ligand	ResName	Monomer	ResNumber	Contact (%)
H27	**VAL**	B	10	96.4
H27	**TYR**	A	9	92.7
H27	**ASP**	B	109	92.2
H27	**TYR**	A	116	88.7
H27	**GLN**	A	118	88.4
H27	**HIS**	B	11	84.9
H27	**VAL**	B	108	84.7
H27	**PRO**	A	121	81.1
H27	**ILE**	B	112	78.9
H27	**ARG**	A	117	67.6
H27	**ILE**	B	5	62.4
H27	**VAL**	A	2	60.2
H27	**GLN**	A	113	52
H27	**GLN**	A	4	51.3
H27	**ILE**	A	112	51.1
H27	**PRO**	A	123	43.1
H27	**TYR**	B	9	42.4
H27	**GLN**	B	3	35.8
H27	**PRO**	B	13	34.4
H27	**HIS**	A	11	33.6
H27	**GLY**	A	7	31.1
H27	**TYR**	B	49	30.9
H27	**LEU**	B	12	30
H27	**ASN**	B	8	29.3
H27	**VAL**	A	10	28.2
H27	**GLN**	B	113	24.7
H27	**MET**	A	115	23.6
H27	**ASN**	A	8	21.6
H27	**VAL**	B	2	17.8
H27	**GLN**	A	3	14.9
H27	**GLY**	B	6	13.8
H27	**ASN**	A	120	10.7
H27	**ASN**	A	126	10.2

C

84-HPVHAGPIAPGQMREPR-100 **HIV-1**

84 85 86 87 88 89 90 91 92 93 94 95 96 97 98 99 100

83-HPQPA–PQ–QGQLREPS-97 **SIVmac**

83 84 85 86 87 88 89 90 91 92 93 94 95 96 97

Appendix Figure S10: Titration on HeLa-R5 cells of PBMC supernatants infected with patient plasma. Infectivity was assessed on HeLa-R5 cells at 48 hpi using undiluted supernatants isolated at the indicated days after PBMC inoculation. β-galactosidase activity is provided as relative light units (RLU) per s. Infectious virus was successfully isolated from the plasma of 7 out of 15 patients. The first supernatant samples yielding an infectivity that was above 1000 RLU/s were selected for experiments shown in **Fig. 6A** to avoid the selection of mutations at later time points. The HIV-1 subtypes were **(A)** Subtype A, **(B)** Subtype B, **(C)** Subtype C, **(D)** Subtype D, **(E)** Subtype F, **(F)** Subtype G, **(G)** Circulating Recombinant Form CRF01_AE and **(H)** CRF02_AG.

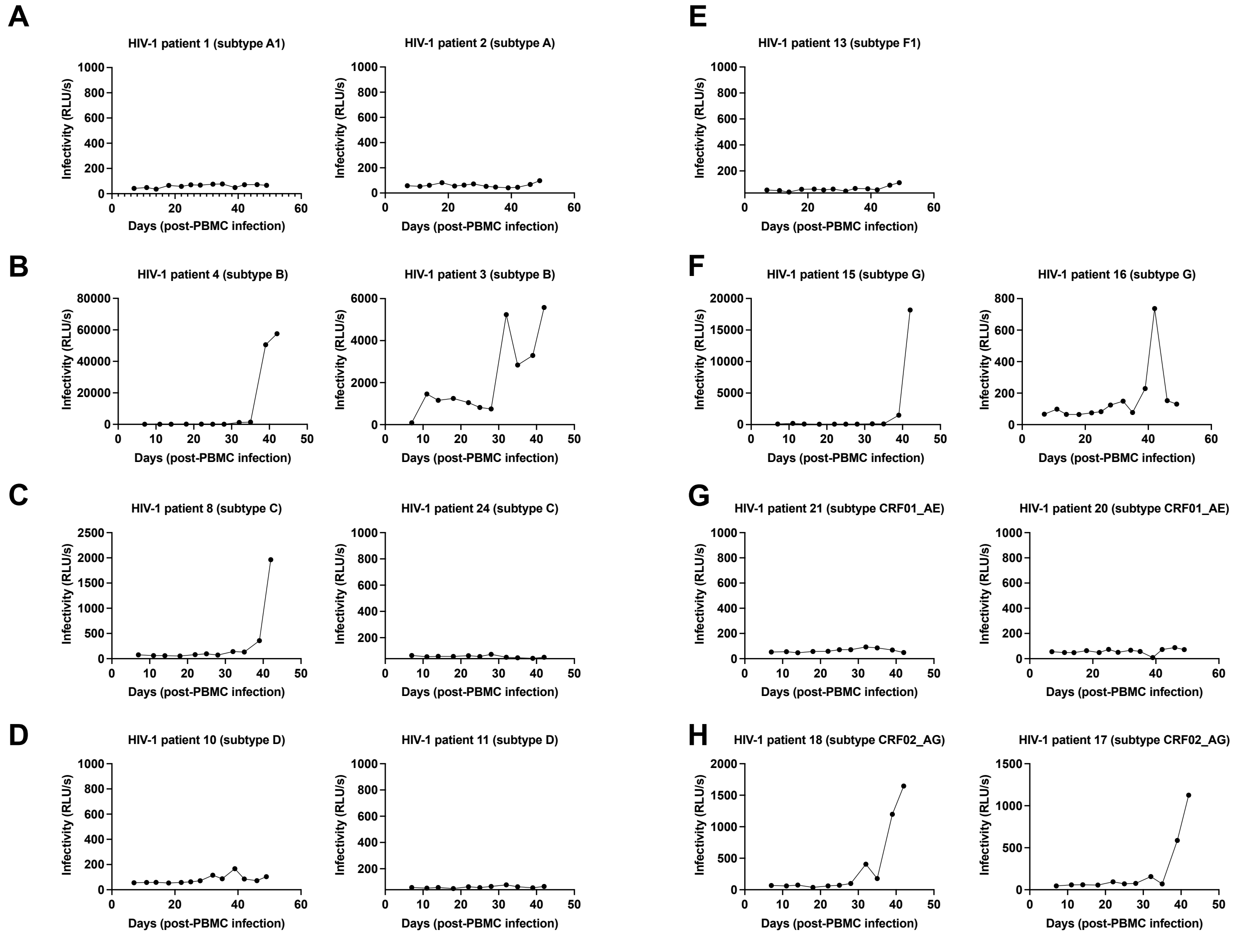

Appendix Figure S11: *In vivo* evaluation of H27 pharmacokinetics and toxicity. (A, B) Pharmacokinetic evaluation of H27 in whole blood after single administration. Male C57BL/6 mice were injected **(A)** intravenously (i.v.) or **(B)** intraperitoneally (i.p.) with H27 at 1 or 3 mg/kg, respectively, with 3 mice per group. Serial blood sampling in the tail was performed at 5, 15, 30 min, then 1, 2, 4, 8 and 12 hours for each mouse following i.v. injection or 15, 30 min, then 1, 2, 4, 8 h following i.p. injection. The $t_{1/2}$ of H27 was 1.6 h after i.v. injection and 5.3 h by i.p. route. Results show the mean values from n = 3 animals at each time point ± SD. **(C, D)** Body weight and clinical scores following administration of H27. Female humanized mice were treated every 3-4 days from D37 to D44 and every 2 days from D48 to D69 with the vehicle (30% Kolliphor/ 70% H2O) or with H27 treatment at 3 mg/kg by intraperitoneal injection. **(C)** Body weight change from D7 to D69. Body weight change is calculated in reference to the body weight measured at D0. (Left) Average body weight change expressed as mean + SEM in % from D7 to D69. A two-way repeated measures ANOVA with Geisser-Greenhouse and Sidak's multiple comparisons tests was used. No statistical difference was observed. (Centre) Individual body weight change of the 6 vehicle treated mice from D7 to D69. (Right) Individual body weight change of the 6 H27 treated mice from D7 to D69. **(D)** Clinical Scores from D7 to D69. (Left) Average clinical score is expressed as mean + SEM from D0 to D69. A two-way repeated measures ANOVA with Geisser– Greenhouse and Sidak's multiple comparisons tests was used. ** p = 0.0088. (Centre) Individual clinical score of the 6 vehicle treated mice from D0 to D69. (Right) Individual clinical score of the 6 H27 treated mice from D0 to D69.

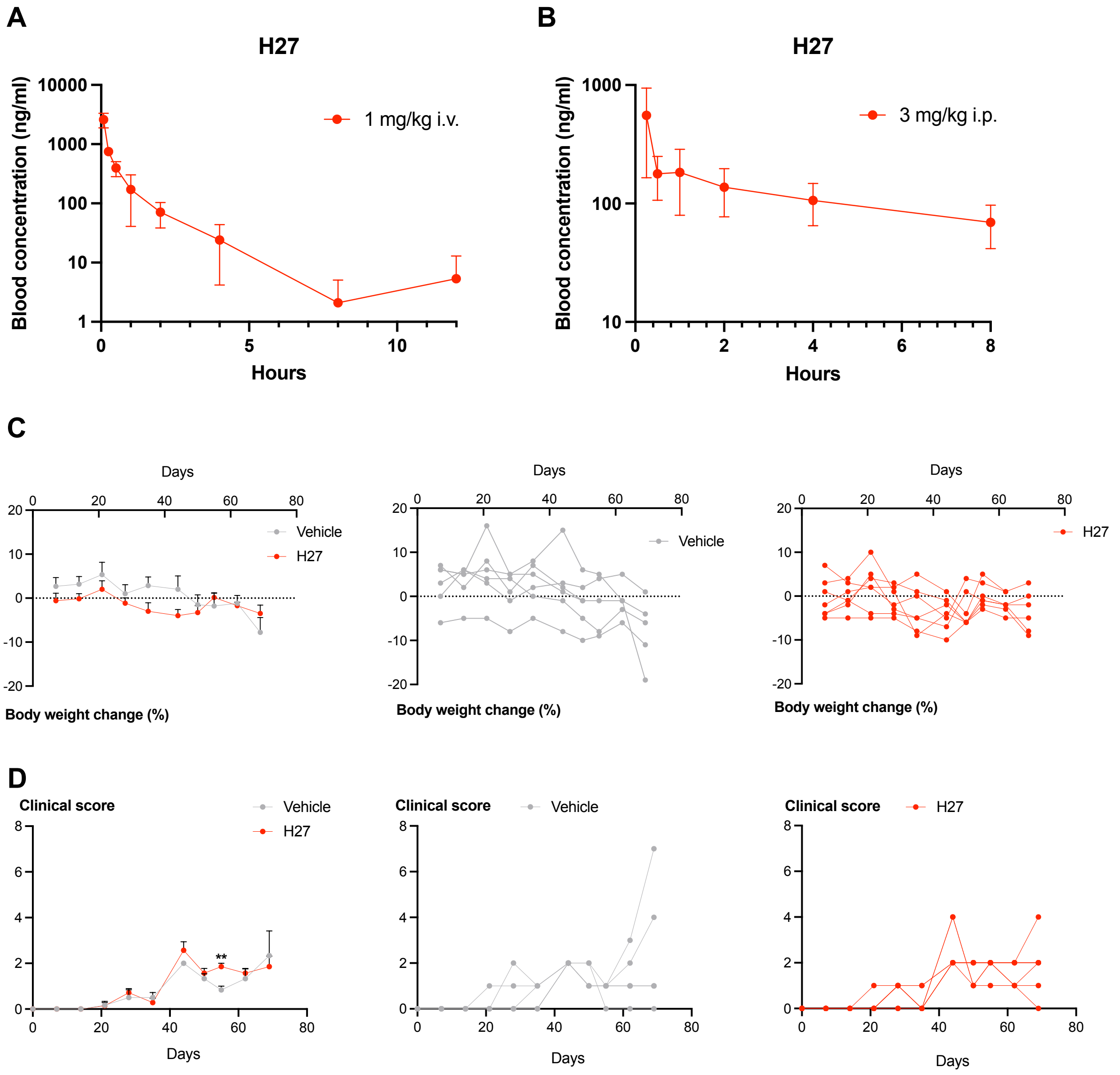
A
H27
1 mg/kg i.v.
Blood concentration (ng/ml)
10000
1000
100
10
1
0
5
10
Hours
B
H27
3 mg/kg i.p.
Blood concentration (ng/ml)
1000
100
10
0
2
4
6
8
Hours
C
Days
Vehicle
H27
Body weight change (%)
Days
Vehicle
Body weight change (%)
Days
H27
Body weight change (%)
D
Clinical score
Vehicle
H27
**
Days
Clinical score
Vehicle
Days
Clinical score
H27
Days

Appendix Table S1: Exact p values for all figures.

Fig. 2G		
Ordinary one-way ANOVA	Exact p value	Summary
H27	0.3369	ns
PF74	0.0002	***
LEN	<0.0001	****

Fig. 5D		
**CA::TRN-1**		
Kruskal-Wallis test	Adjusted p value	Summary
DMSO vs. ni	0.0016	**
DMSO vs. H27	0.0322	*
DMSO vs. PF74	0.5406	ns
DMSO vs. LEN	>0.9999	ns
**CA::CPSF6**		
Kruskal-Wallis test	Adjusted p value	Summary
DMSO vs. ni	0.0004	***
DMSO vs. H27	>0.9999	ns
DMSO vs. PF74	>0.9999	ns
DMSO vs. LEN	0.0155	*

Fig. 6A		
**YU2**		
RM one-way ANOVA	Adjusted P value	Summary
DMSO vs. 5 µM	<0.0001	****
DMSO vs. 10 µM	<0.0001	****
DMSO vs. 50 µM	<0.0001	****
DMSO vs. NVP	<0.0001	****
DMSO vs. 3TC	<0.0001	****
**Patient 3**		
RM one-way ANOVA	Adjusted P value	Summary
DMSO vs. 5 µM	<0.0001	****
DMSO vs. 10 µM	<0.0001	****
DMSO vs. 50 µM	<0.0001	****
DMSO vs. NVP	<0.0001	****
DMSO vs. 3TC	<0.0001	****
**Patient 4**		
RM one-way ANOVA	Adjusted P value	Summary
DMSO vs. 5 µM	0.0160	*
DMSO vs. 10 µM	0.0007	***
DMSO vs. 50 µM	<0.0001	****
DMSO vs. NVP	<0.0001	****
DMSO vs. 3TC	<0.0001	****
**Patient 8**		
RM one-way ANOVA	Adjusted P value	Summary
DMSO vs. 5 µM	0.2085	ns
DMSO vs. 10 µM	0.0086	**
DMSO vs. 50 µM	<0.0001	****
DMSO vs. NVP	<0.0001	****
DMSO vs. 3TC	<0.0001	****
**Patient 10**		
RM one-way ANOVA	Adjusted P value	Summary
DMSO vs. 5 µM	0.0223	*
DMSO vs. 10 µM	<0.0001	****

DMSO vs. 50 µM	<0.0001	****
DMSO vs. NVP	<0.0001	****
DMSO vs. 3TC	<0.0001	****
**Patient 15**		
RM one-way ANOVA	Adjusted P value	Summary
DMSO vs. 5 µM	<0.0001	****
DMSO vs. 10 µM	<0.0001	****
DMSO vs. 50 µM	<0.0001	****
DMSO vs. NVP	<0.0001	****
DMSO vs. 3TC	<0.0001	****
**Patient 17**		
RM one-way ANOVA	Adjusted P value	Summary
DMSO vs. 5 µM	0.0001	***
DMSO vs. 10 µM	<0.0001	****
DMSO vs. 50 µM	<0.0001	****
DMSO vs. NVP	<0.0001	****
DMSO vs. 3TC	<0.0001	****
**Patient 18**		
RM one-way ANOVA	Adjusted P value	Summary
DMSO vs. 5 µM	<0.0001	****
DMSO vs. 10 µM	<0.0001	****
DMSO vs. 50 µM	<0.0001	****
DMSO vs. NVP	<0.0001	****
DMSO vs. 3TC	<0.0001	****

Fig. 6B		
**NL4-3**		
RM one-way ANOVA	Adjusted P value	Summary
DMSO vs. 5 μM	0.0994	ns
DMSO vs. 10 μM	<0.0001	****
DMSO vs. 50 μM	<0.0001	****
DMSO vs. 3TC	<0.0001	****
DMSO vs. NVP	<0.0001	****
**NL4-3 R263K**		
RM one-way ANOVA	Adjusted P value	Summary
DMSO vs. 5 μM	0.0095	**
DMSO vs. 10 μM	0.0007	***
DMSO vs. 50 μM	0.0002	***
DMSO vs. DTG	0.1888	ns
DMSO vs. NVP	0.0002	***
**NL4-3 G140S-Q148H**		
RM one-way ANOVA	Adjusted P value	Summary
DMSO vs. 5 μM	0.0030	**
DMSO vs. 10 μM	0.0003	***
DMSO vs. 50 μM	0.0002	***
DMSO vs. RAL	0.4917	ns
DMSO vs. NVP	0.0001	***
**NL4-3 N155H-K211R-E212T**		
RM one-way ANOVA	Adjusted P value	Summary
DMSO vs. 5 μM	0.0010	***
DMSO vs. 10 μM	0.0002	***
DMSO vs. 50 μM	0.0001	***
DMSO vs. RAL	0.9893	ns
DMSO vs. NVP	0.0001	***
**NL4-3 I50V**		
RM one-way ANOVA	Adjusted P value	Summary
DMSO vs. 5 μM	0.3743	ns
DMSO vs. 10 μM	0.0133	*

DMSO vs. 50 µM	0.0013	**
DMSO vs. DRV	0.9266	ns
DMSO vs. NVP	0.0016	**
**NL4-3 V32I-L33F-I47V**		
RM one-way ANOVA	Adjusted P value	Summary
DMSO vs. 5 µM	0.0003	***
DMSO vs. 10 µM	<0.0001	****
DMSO vs. 50 µM	0.0001	***
DMSO vs. DRV	0.3642	ns
DMSO vs. NVP	0.0001	***
**NL4-3 V106M**		
RM one-way ANOVA	Adjusted P value	Summary
DMSO vs. 5 µM	0.0414	*
DMSO vs. 10 µM	0.0072	**
DMSO vs. 50 µM	0.0008	***
DMSO vs. NVP	0.9028	ns
DMSO vs. 3TC	0.0058	**
**NL4-3 M184V**		
RM one-way ANOVA	Adjusted P value	Summary
DMSO vs. 5 µM	0.0011	**
DMSO vs. 10 µM	0.0009	***
DMSO vs. 50 µM	0.0007	***
DMSO vs. 3TC	0.1074	ns
DMSO vs. NVP	0.0009	***

Fig. EV2A		
Wilcoxon test	Exact p value	Summary
Input 2h	0.7500	ns
Cores 2h	0.2500	ns
Friedman test	Exact p value	Summary
Input 16h	0.9444	ns
Cores 16h	0.0046	**

Fig. EV2B		
Ordinary one-way ANOVA	Adjusted P Value	Summary
DMSO vs. H27	0.0028	**
DMSO vs. 31	0.9415	ns
