# Supplementary material for: A new class of capsid-targeting inhibitors that specifically block HIV-1 nuclear import
Source: EMBO Mol Med. 2024 Oct 2;16(11):13. doi: 10.1038/s44321-024-00143-w (PMC11555092; doi:10.1038/s44321-024-00143-w)
Supplement: Supplementary file 9 — Movie EV1 [file 44321_2024_143_MOESM9_ESM.zip › Movie EV1.rtf]

Movie EV1: H27 binding to CA hexamer as predicted by MD simulations. The movie based on the stripped trajectory (1 frame out of 10) of the all-atom simulation performed in explicit water during 0.5 µs. H27 (depicted in cyan balls & sticks) initially located on the upper NTD surface rapidly moved near the PR loop (depicted in thin sticks) and remained bound at the interface between two CA monomers (green and blue cartoon representations) for the remaining simulation time. 
