# Supplementary figures and images for: A new class of capsid-targeting inhibitors that specifically block HIV-1 nuclear import
Source: EMBO Mol Med. 2024 Oct 2;16(11):13. doi: 10.1038/s44321-024-00143-w (PMC11555092; doi:10.1038/s44321-024-00143-w)

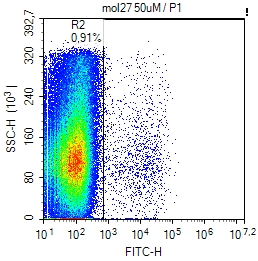

Supplement: Supplementary file 10 — Source data Fig. 1 [file 44321_2024_143_MOESM10_ESM.zip › Figure 1/1F/H27 50.jpg]

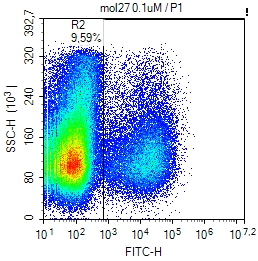

Supplement: Supplementary file 10 — Source data Fig. 1 [file 44321_2024_143_MOESM10_ESM.zip › Figure 1/1F/H27 0_1.jpg]

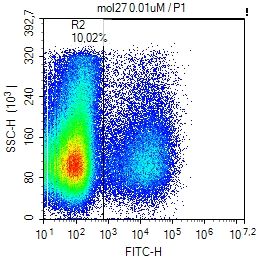

Supplement: Supplementary file 10 — Source data Fig. 1 [file 44321_2024_143_MOESM10_ESM.zip › Figure 1/1F/H27 0_01.jpg]

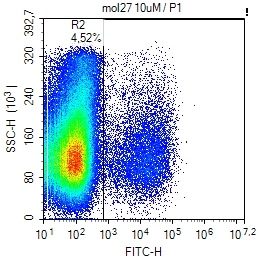

Supplement: Supplementary file 10 — Source data Fig. 1 [file 44321_2024_143_MOESM10_ESM.zip › Figure 1/1F/H27 10.jpg]

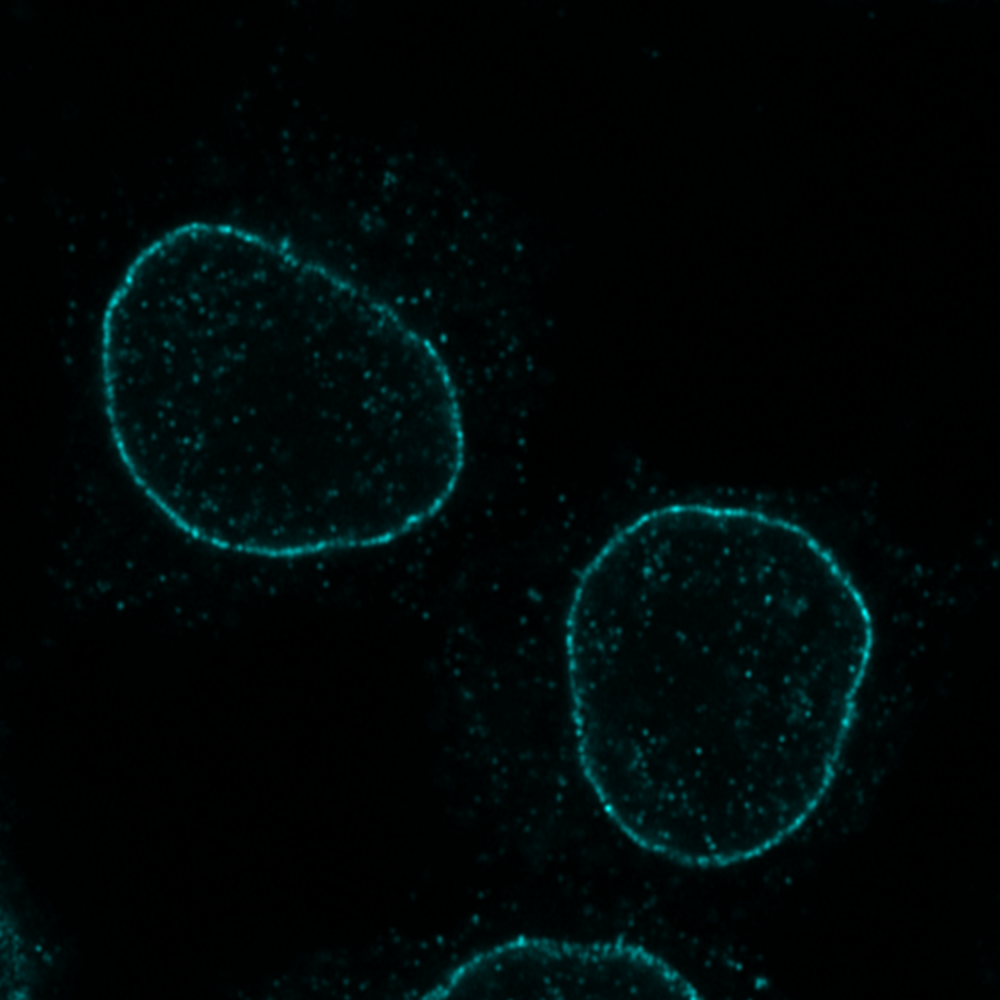

Supplement: Supplementary file 11 — Source data Fig. 2 [file 44321_2024_143_MOESM11_ESM.zip › Figure 2/2F/H27 Nup214.tif]

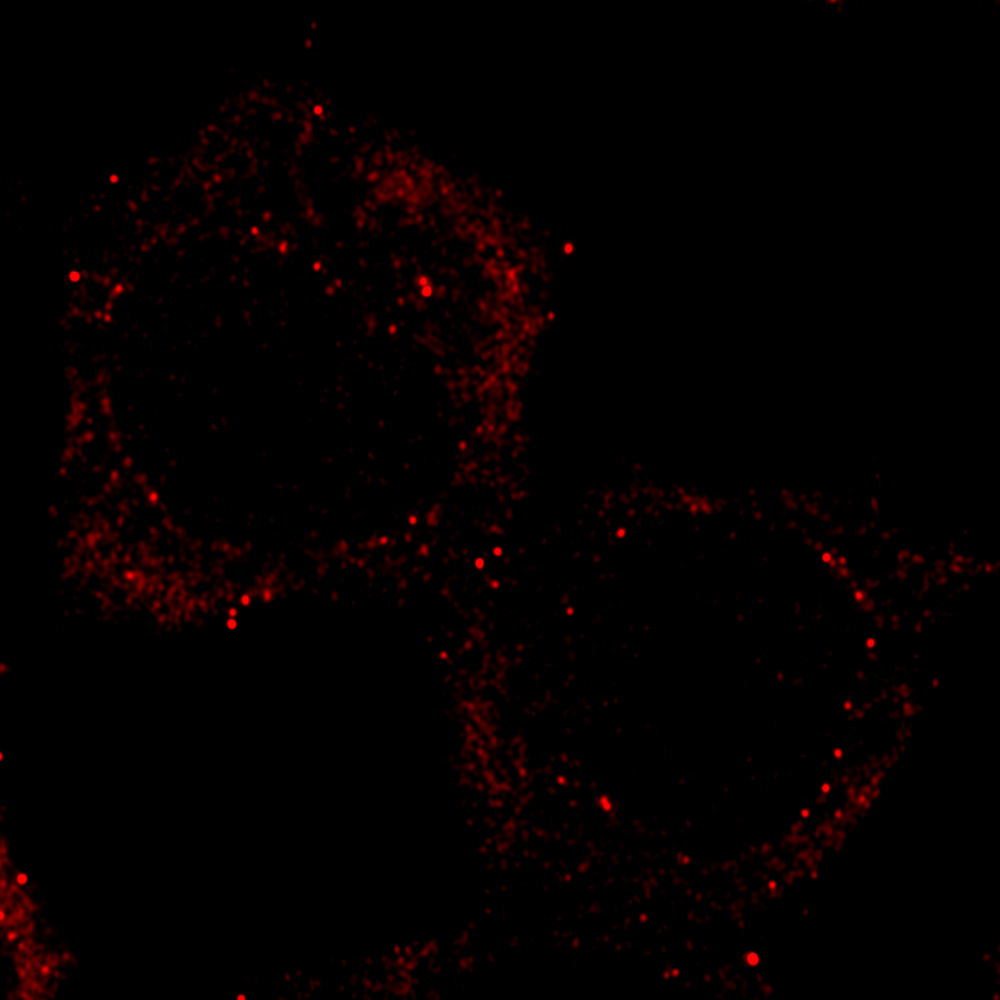

Supplement: Supplementary file 11 — Source data Fig. 2 [file 44321_2024_143_MOESM11_ESM.zip › Figure 2/2F/H27 CA.tif]

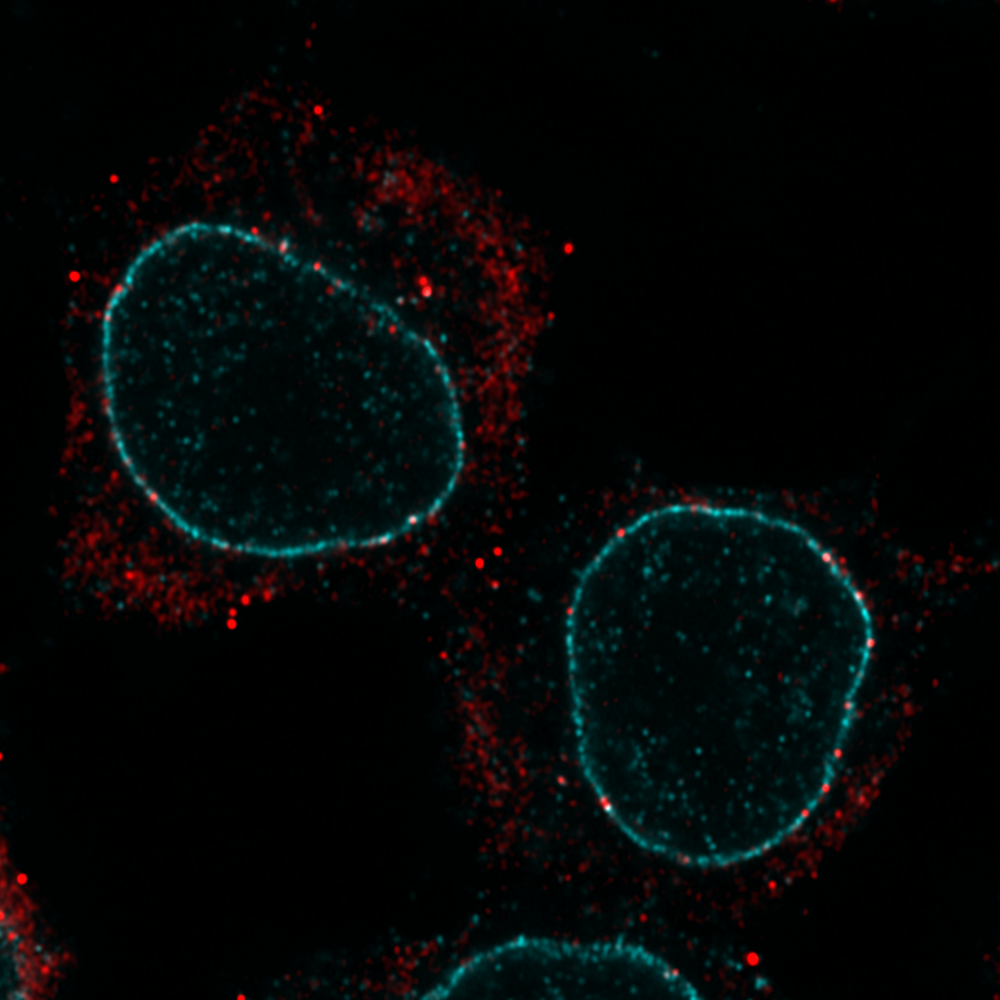

Supplement: Supplementary file 11 — Source data Fig. 2 [file 44321_2024_143_MOESM11_ESM.zip › Figure 2/2F/H27 merge.tif]

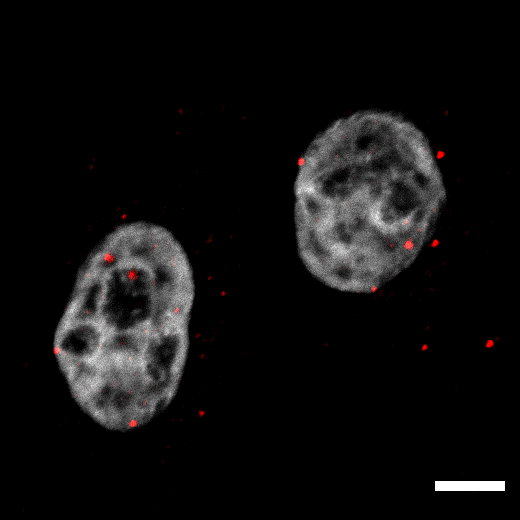

Supplement: Supplementary file 11 — Source data Fig. 2 [file 44321_2024_143_MOESM11_ESM.zip › Figure 2/2D/Composite 40.tif]

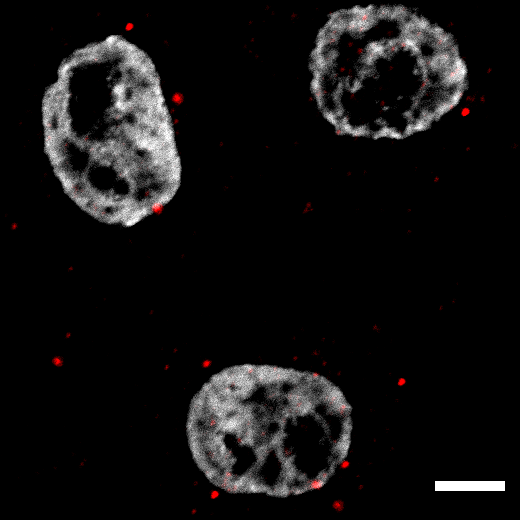

Supplement: Supplementary file 11 — Source data Fig. 2 [file 44321_2024_143_MOESM11_ESM.zip › Figure 2/2D/Composite H27.tif]

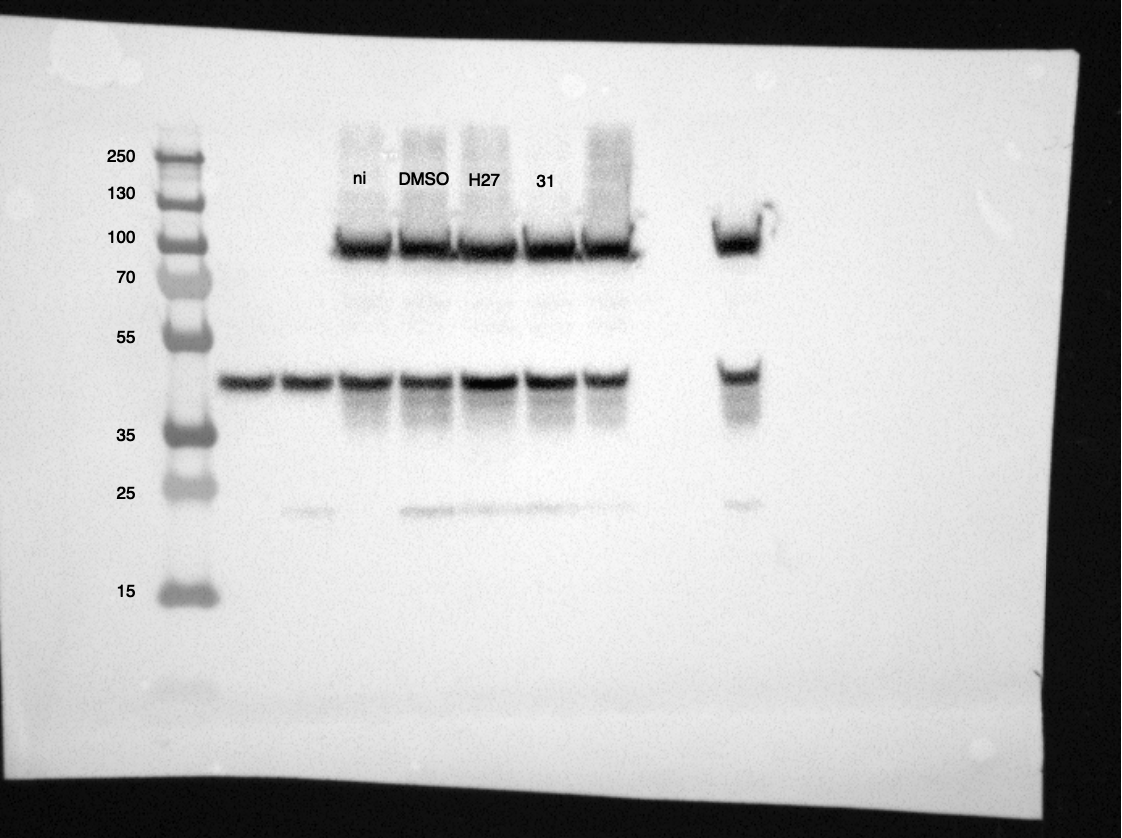

Supplement: Supplementary file 14 — Source data Fig. 5 [file 44321_2024_143_MOESM14_ESM.zip › Figure 5/5A/5A Input HA western.tif]

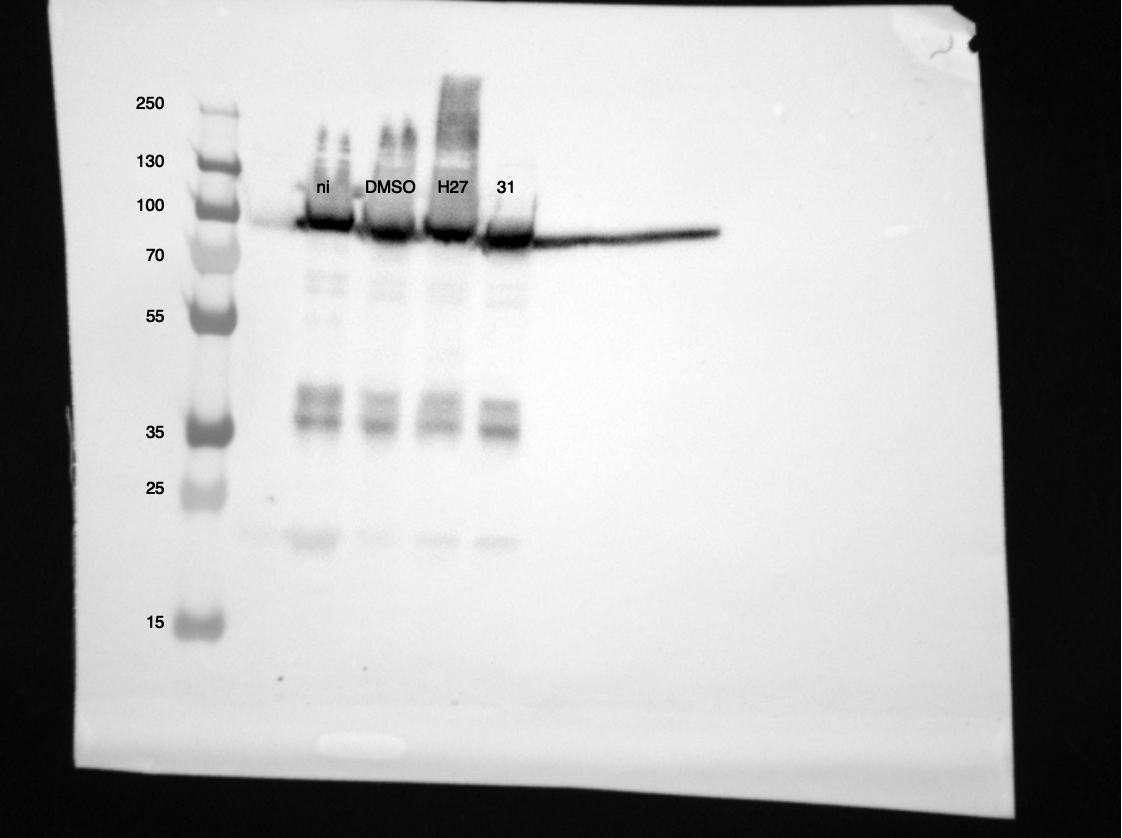

Supplement: Supplementary file 14 — Source data Fig. 5 [file 44321_2024_143_MOESM14_ESM.zip › Figure 5/5A/5A IP HA western.tif]

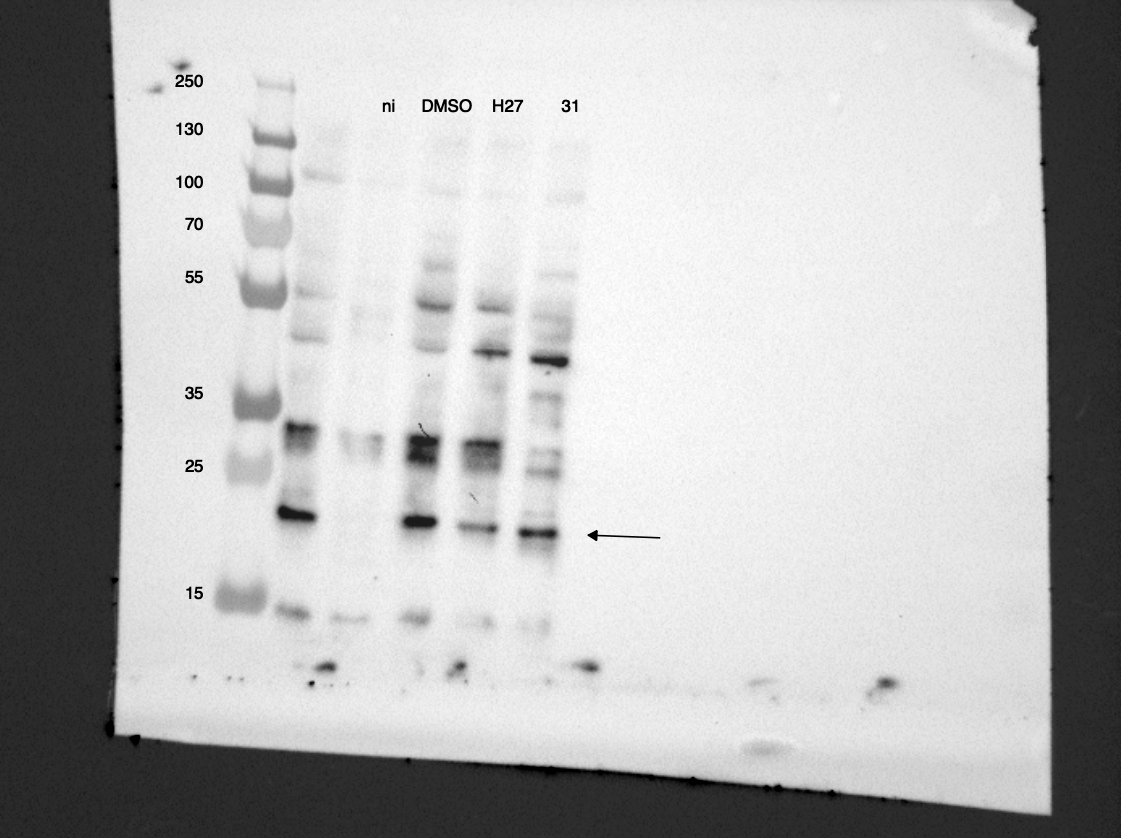

Supplement: Supplementary file 14 — Source data Fig. 5 [file 44321_2024_143_MOESM14_ESM.zip › Figure 5/5A/5A IP p24 western.tif]

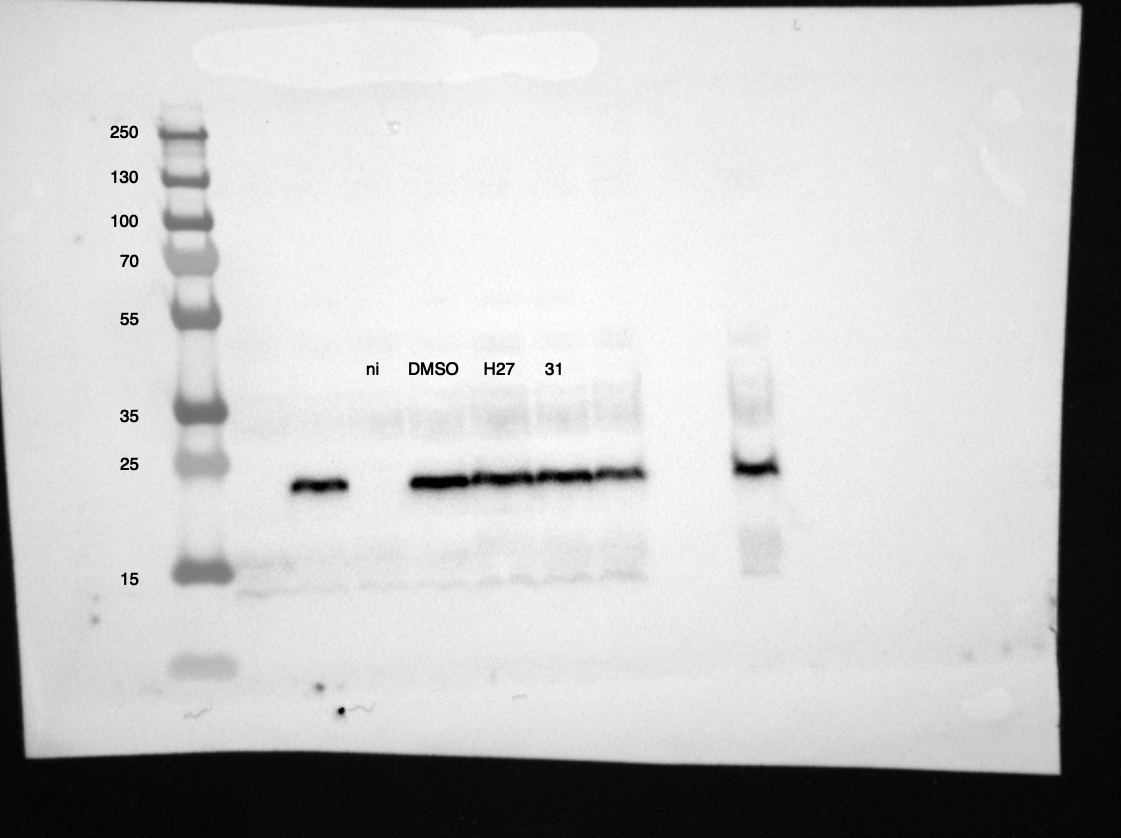

Supplement: Supplementary file 14 — Source data Fig. 5 [file 44321_2024_143_MOESM14_ESM.zip › Figure 5/5A/5A Input p24 western.tif]

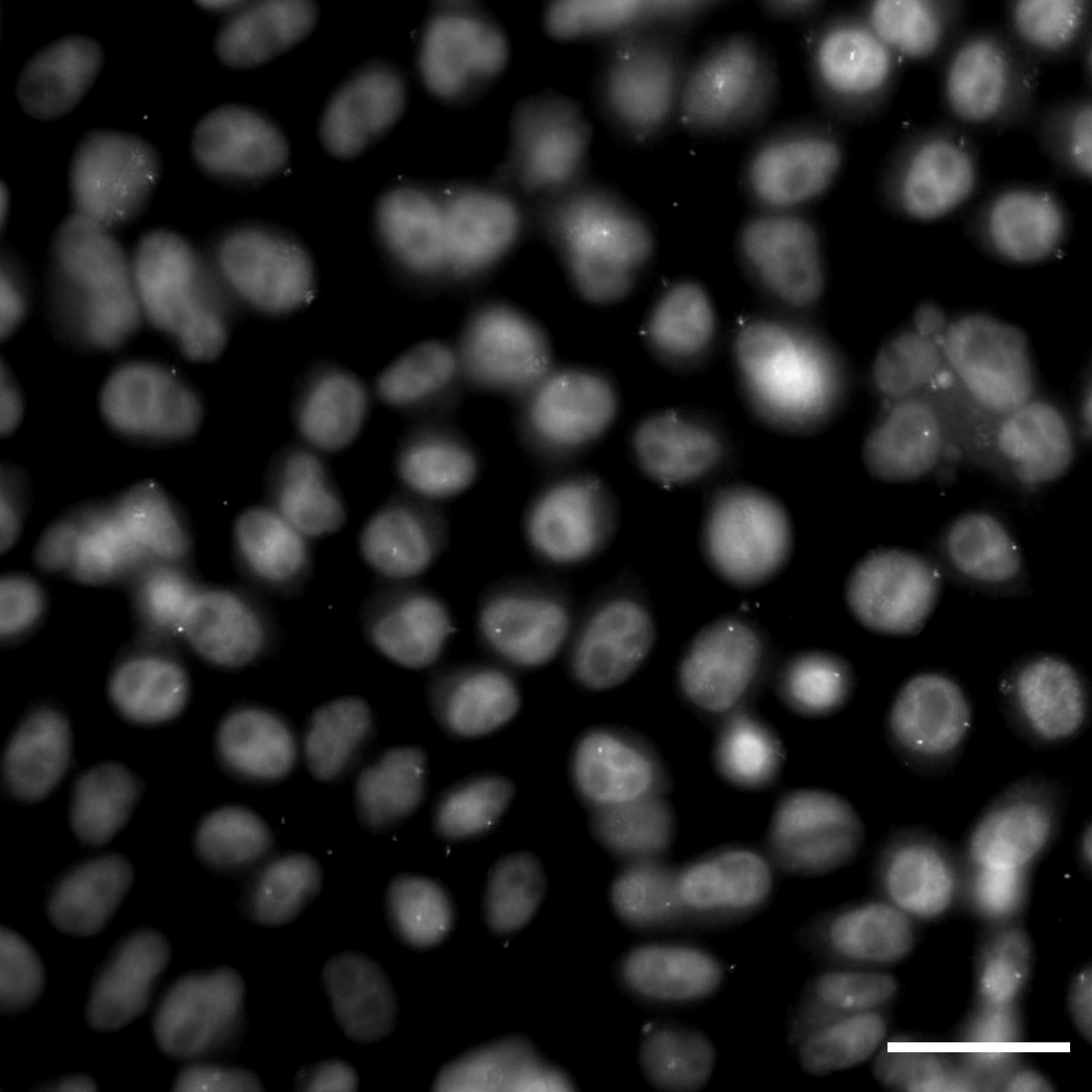

Supplement: Supplementary file 14 — Source data Fig. 5 [file 44321_2024_143_MOESM14_ESM.zip › Figure 5/5D/catrn h27.tif]

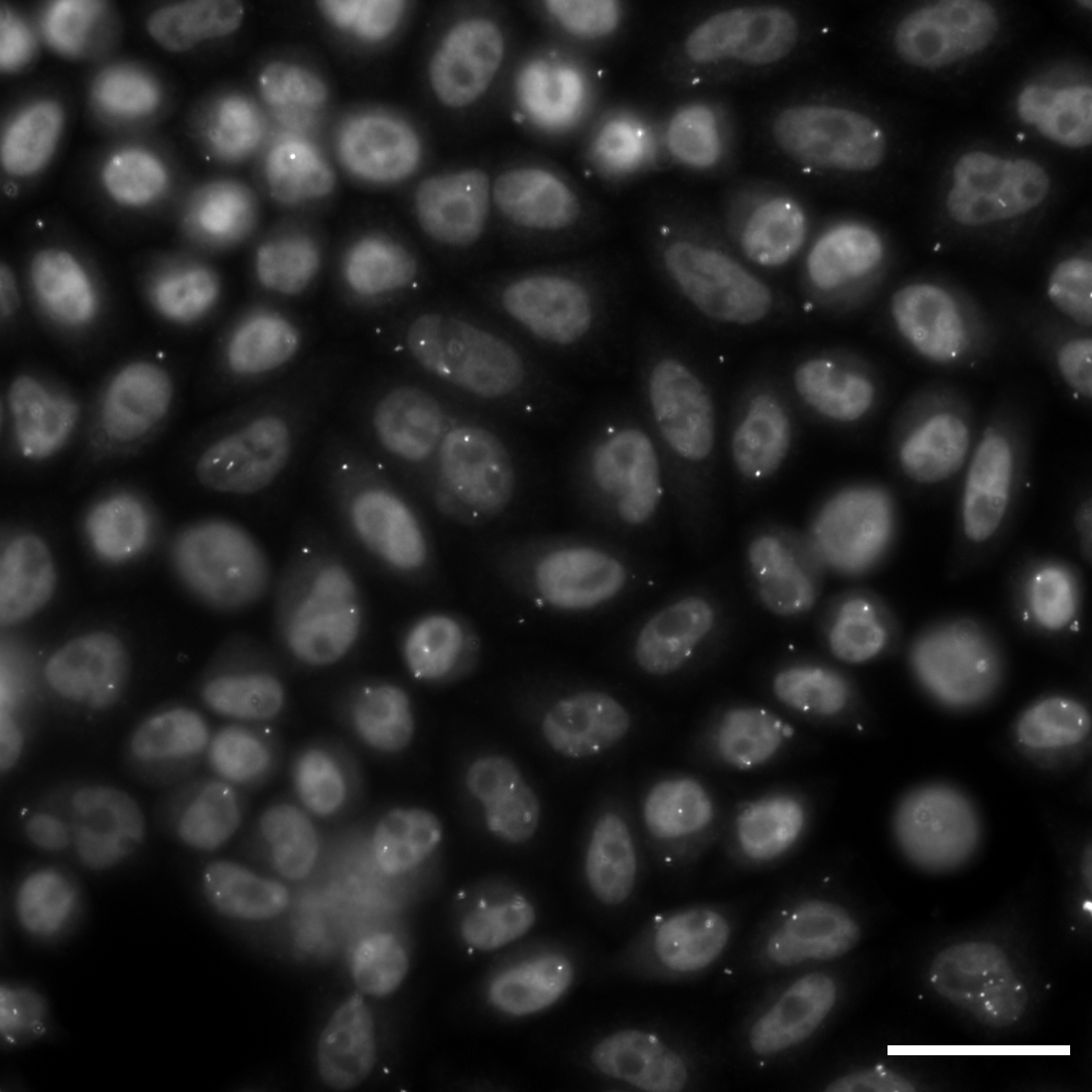

Supplement: Supplementary file 14 — Source data Fig. 5 [file 44321_2024_143_MOESM14_ESM.zip › Figure 5/5D/catrn len.tif]

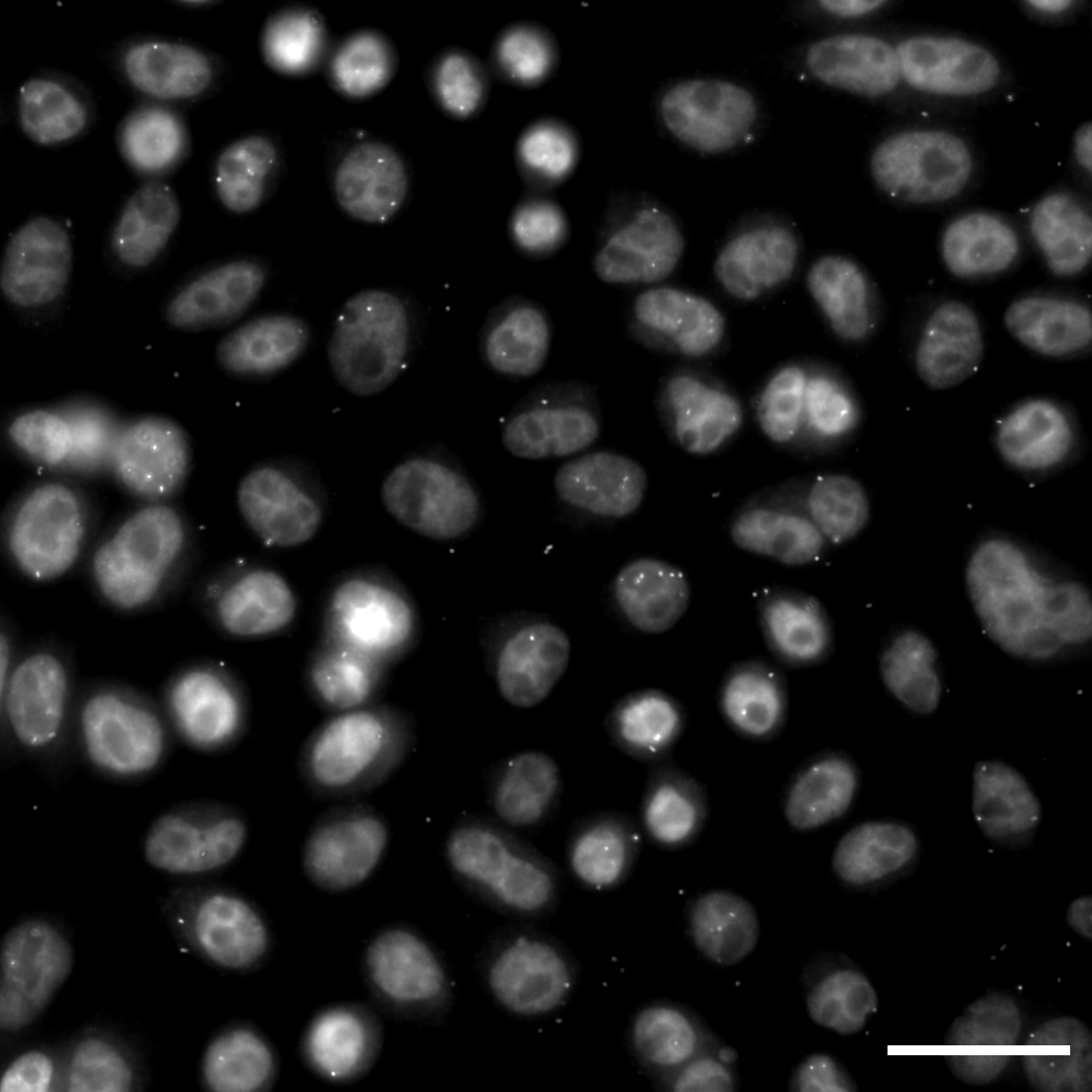

Supplement: Supplementary file 14 — Source data Fig. 5 [file 44321_2024_143_MOESM14_ESM.zip › Figure 5/5D/catrn pf74.tif]

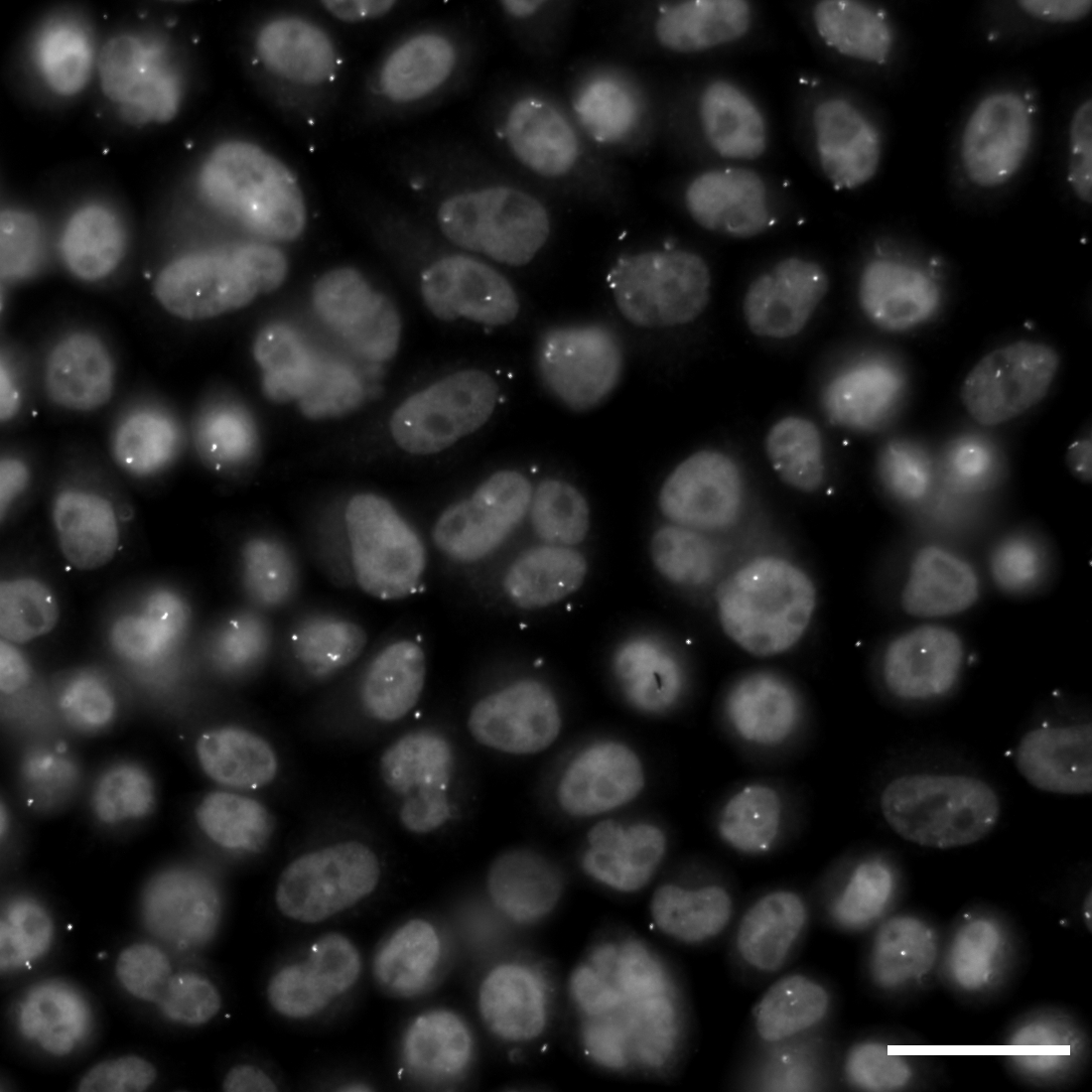

Supplement: Supplementary file 14 — Source data Fig. 5 [file 44321_2024_143_MOESM14_ESM.zip › Figure 5/5D/catrn dmso.tif]

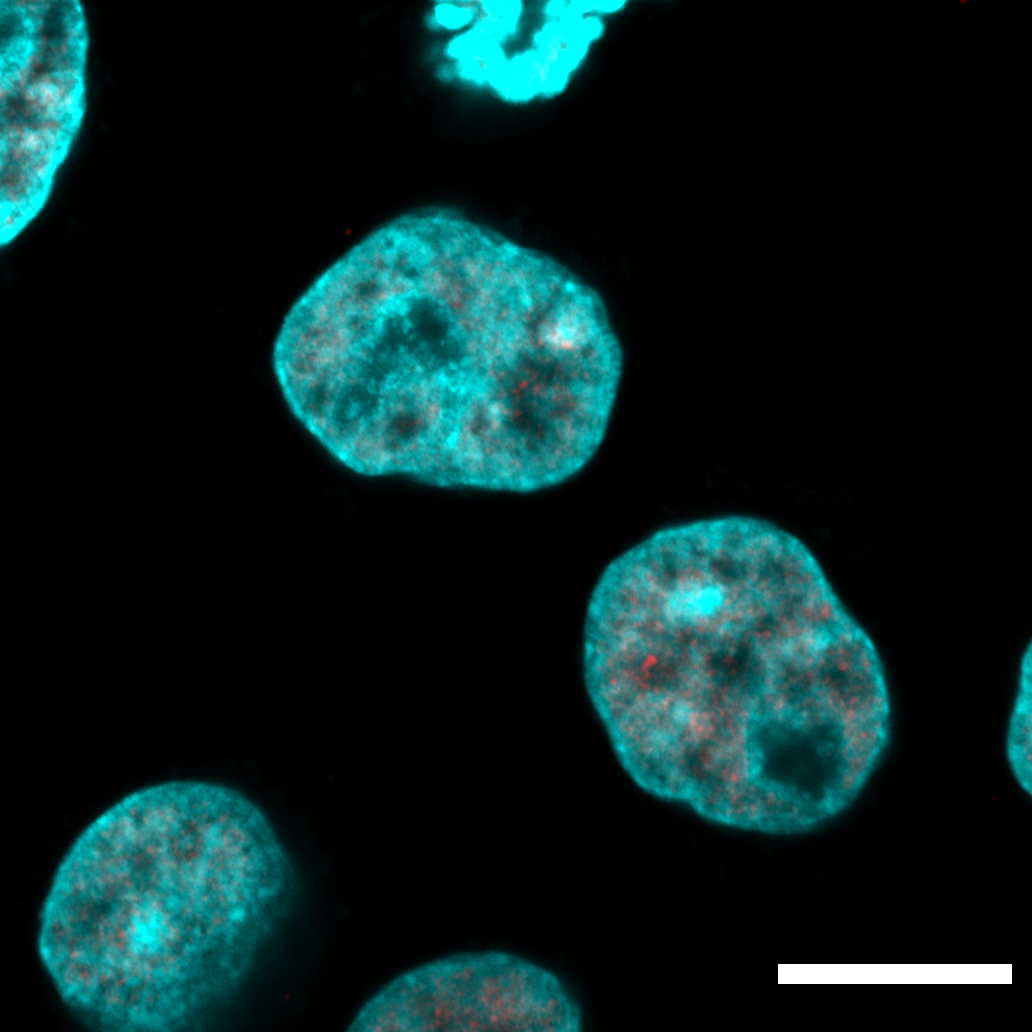

Supplement: Supplementary file 14 — Source data Fig. 5 [file 44321_2024_143_MOESM14_ESM.zip › Figure 5/5E/Composite H27.tif]

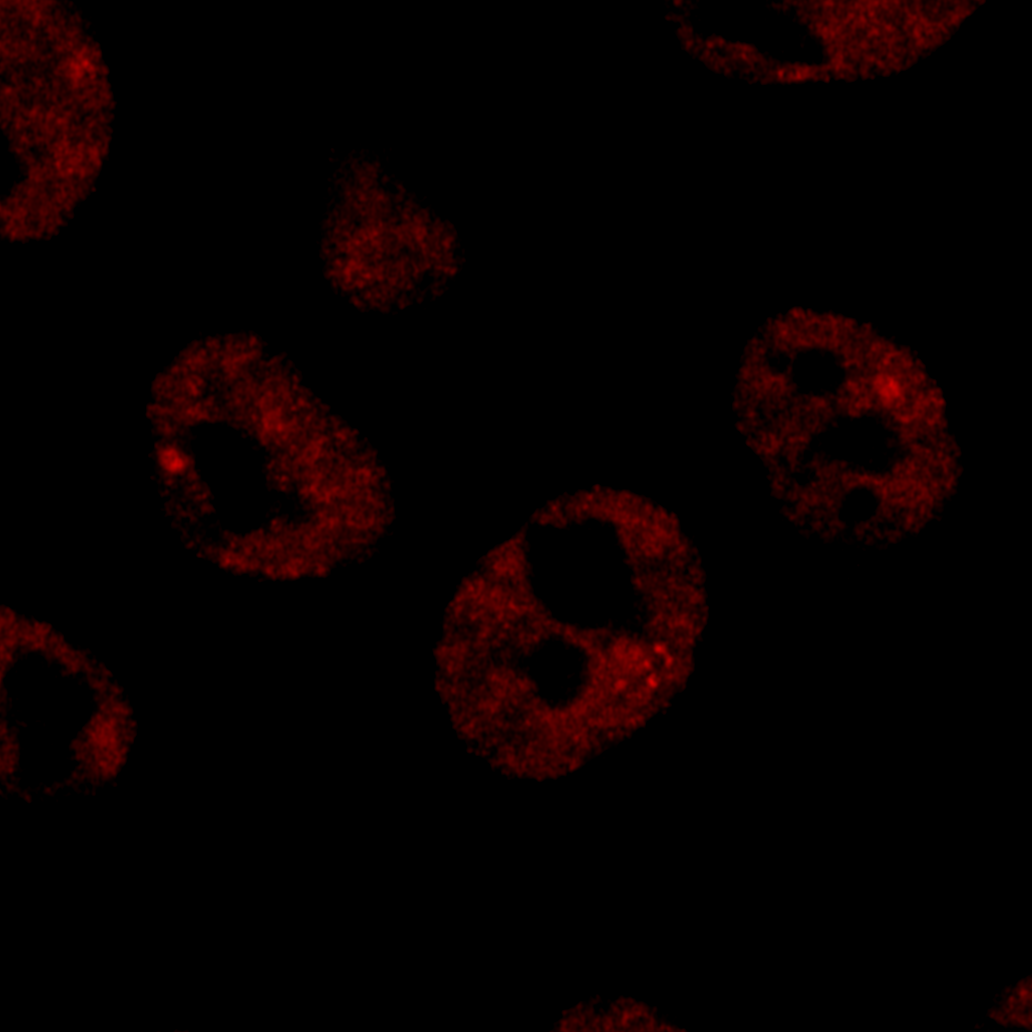

Supplement: Supplementary file 14 — Source data Fig. 5 [file 44321_2024_143_MOESM14_ESM.zip › Figure 5/5E/PLA ni.tif]

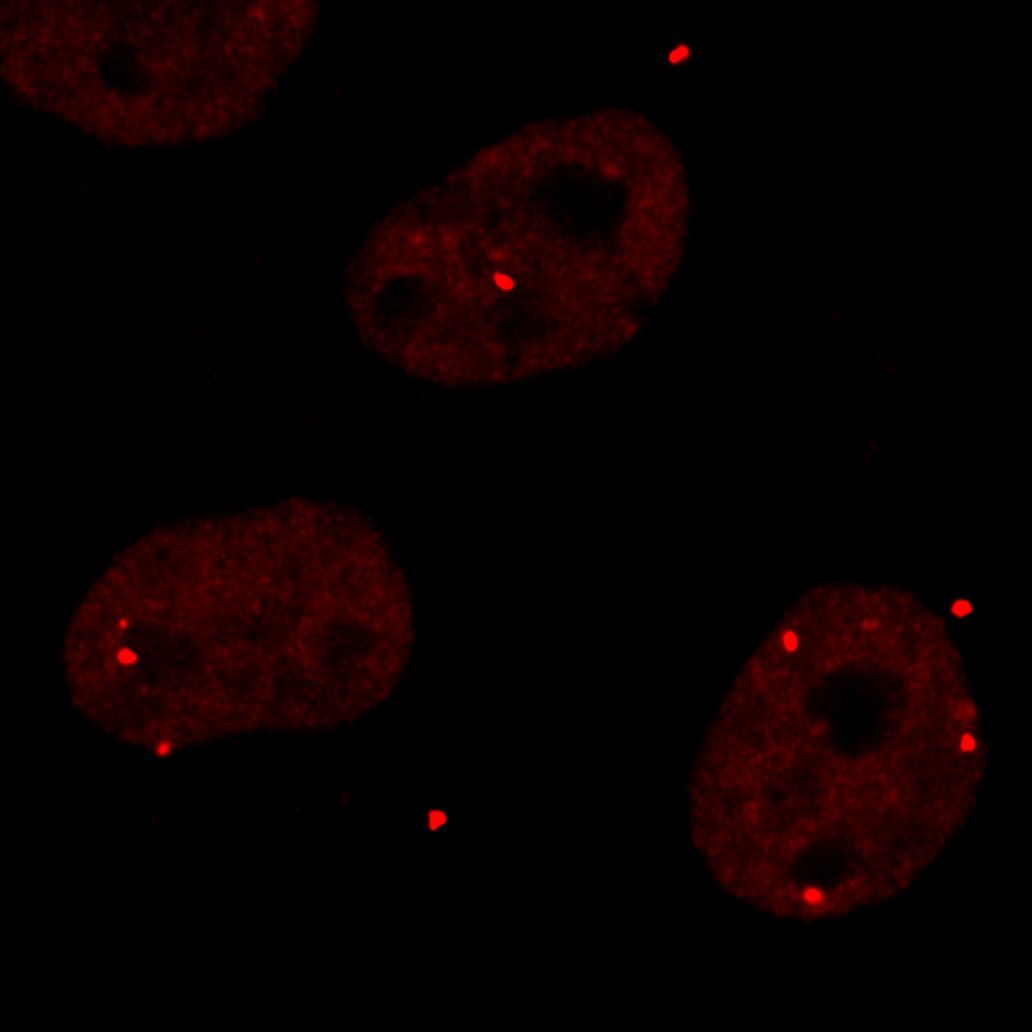

Supplement: Supplementary file 14 — Source data Fig. 5 [file 44321_2024_143_MOESM14_ESM.zip › Figure 5/5E/PLA LEN.tif]

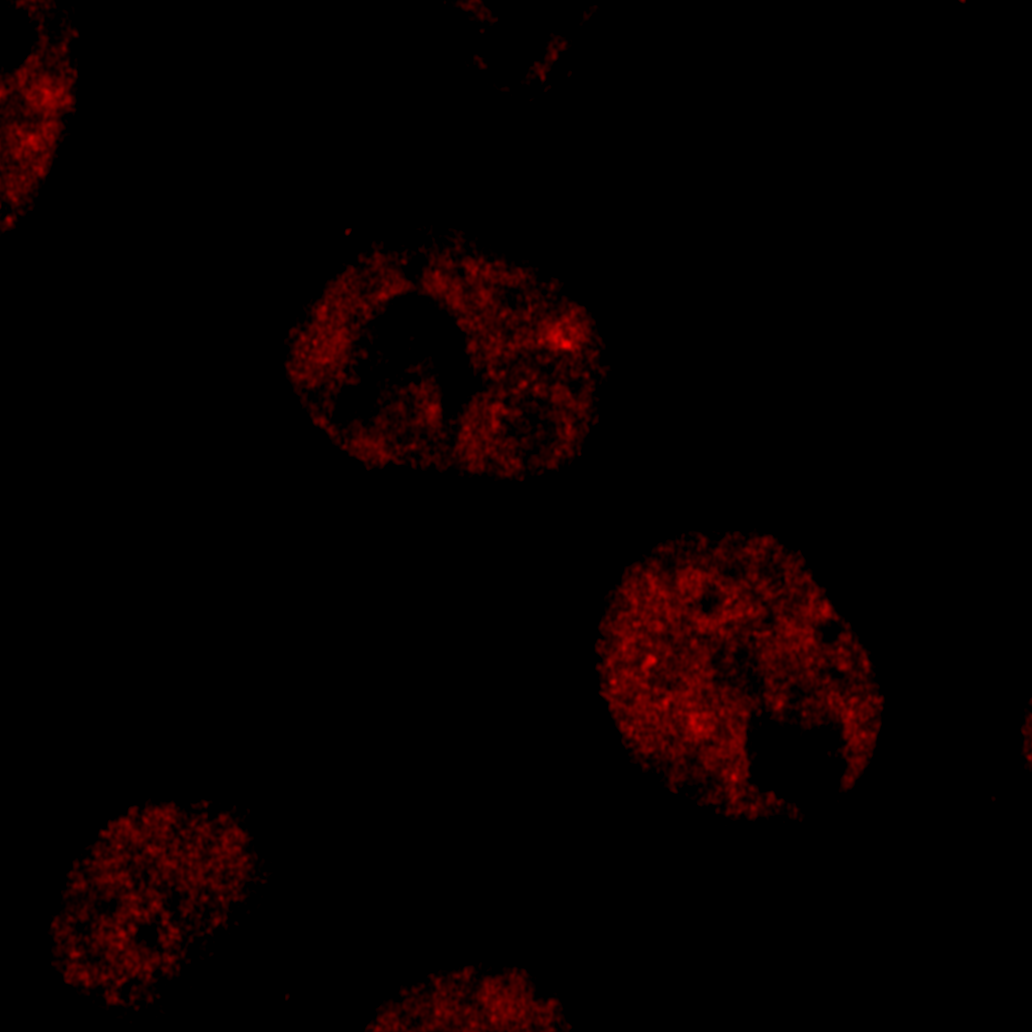

Supplement: Supplementary file 14 — Source data Fig. 5 [file 44321_2024_143_MOESM14_ESM.zip › Figure 5/5E/PLA H27.tif]

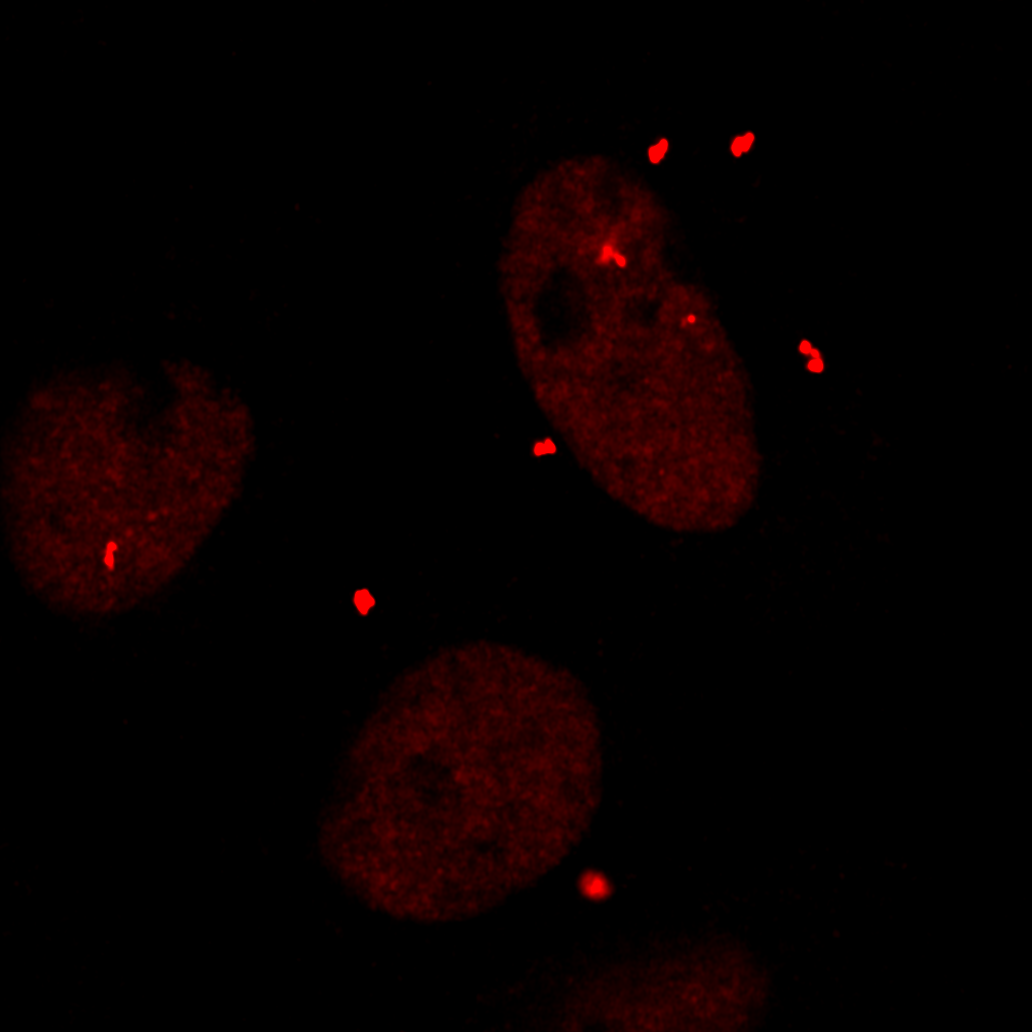

Supplement: Supplementary file 14 — Source data Fig. 5 [file 44321_2024_143_MOESM14_ESM.zip › Figure 5/5E/PLA DMSO.tif]

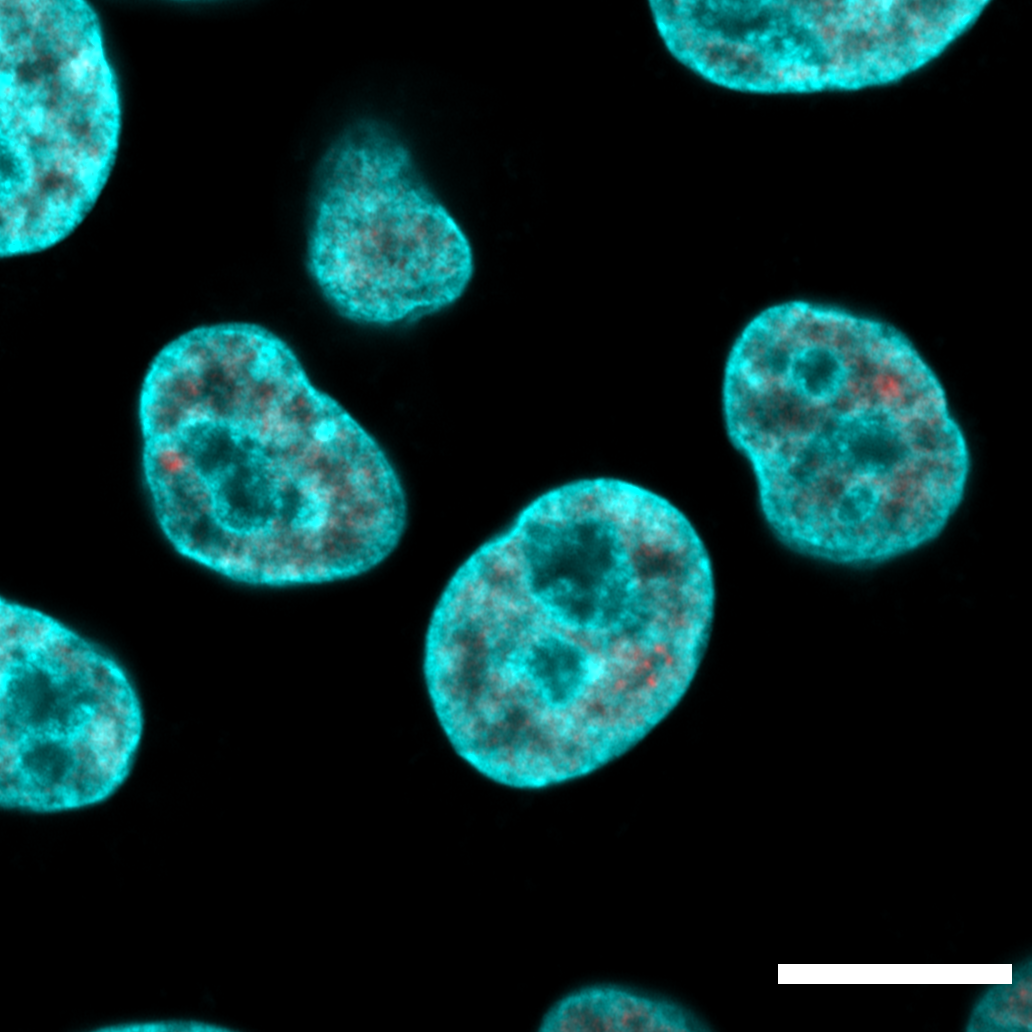

Supplement: Supplementary file 14 — Source data Fig. 5 [file 44321_2024_143_MOESM14_ESM.zip › Figure 5/5E/Composite ni.tif]

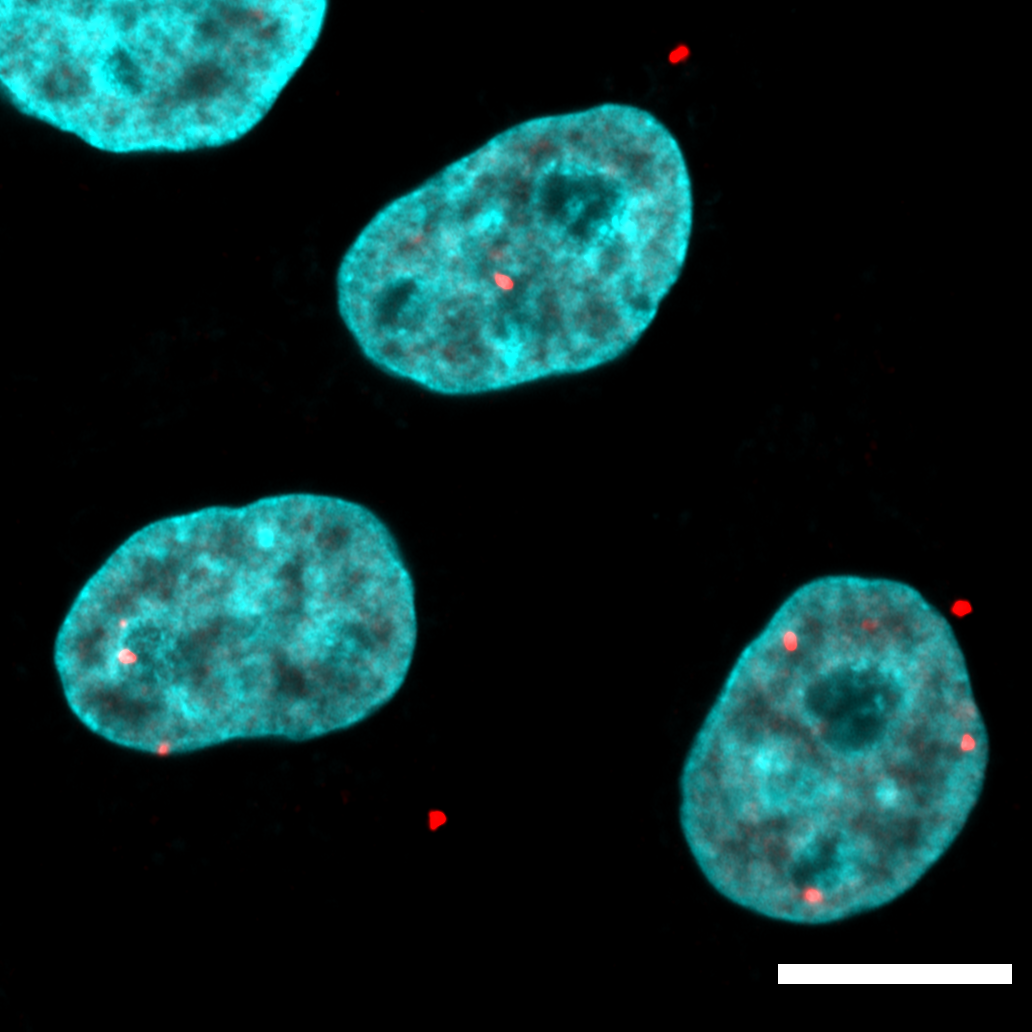

Supplement: Supplementary file 14 — Source data Fig. 5 [file 44321_2024_143_MOESM14_ESM.zip › Figure 5/5E/Composite LEN.tif]

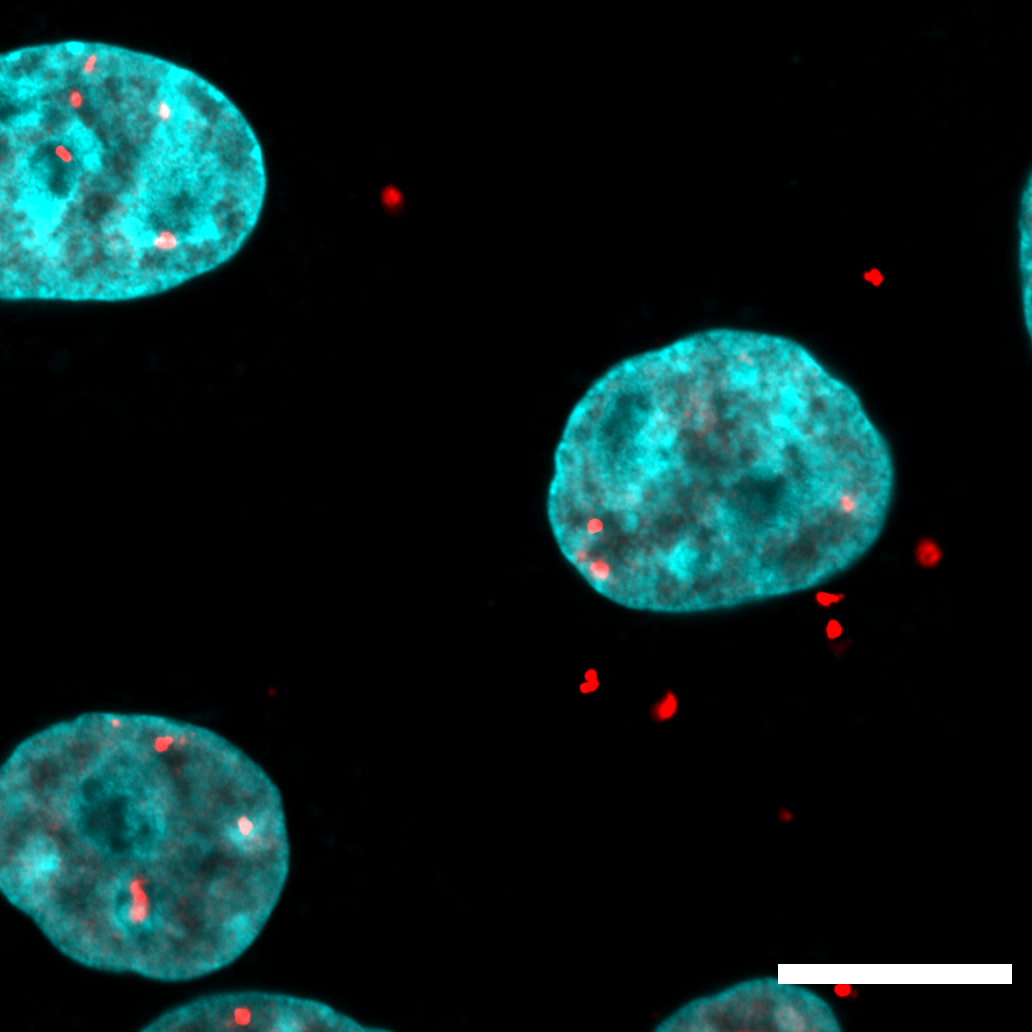

Supplement: Supplementary file 14 — Source data Fig. 5 [file 44321_2024_143_MOESM14_ESM.zip › Figure 5/5E/Composite PF74.tif]

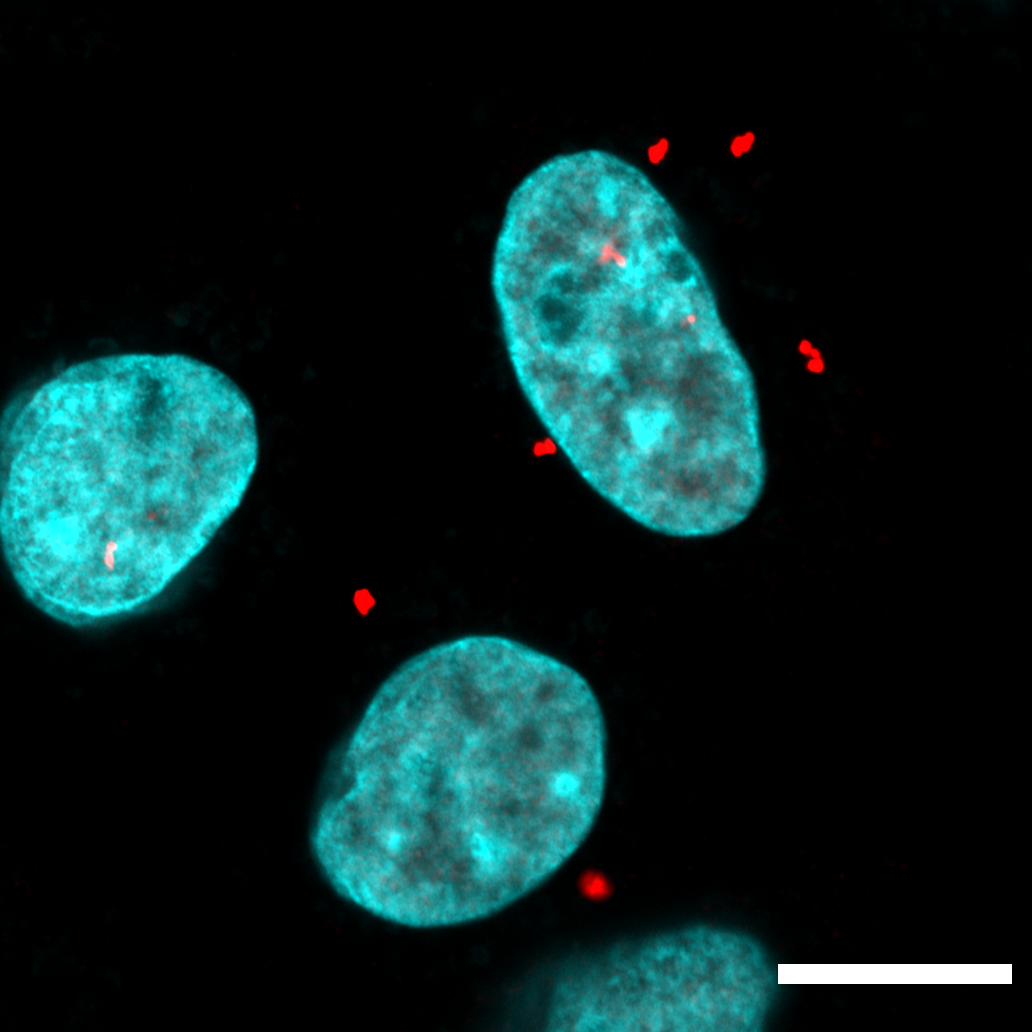

Supplement: Supplementary file 14 — Source data Fig. 5 [file 44321_2024_143_MOESM14_ESM.zip › Figure 5/5E/Composite DMSO.tif]

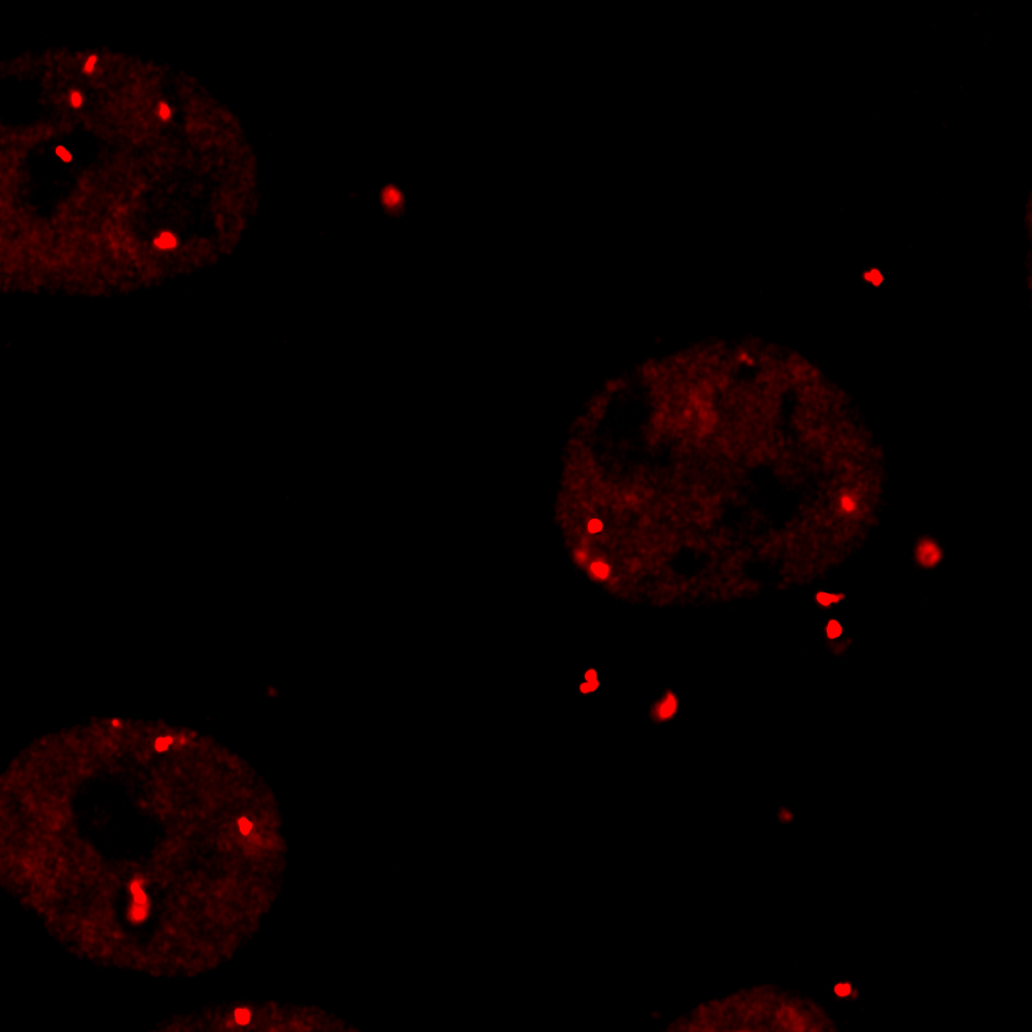

Supplement: Supplementary file 14 — Source data Fig. 5 [file 44321_2024_143_MOESM14_ESM.zip › Figure 5/5E/PLA PF74.tif]

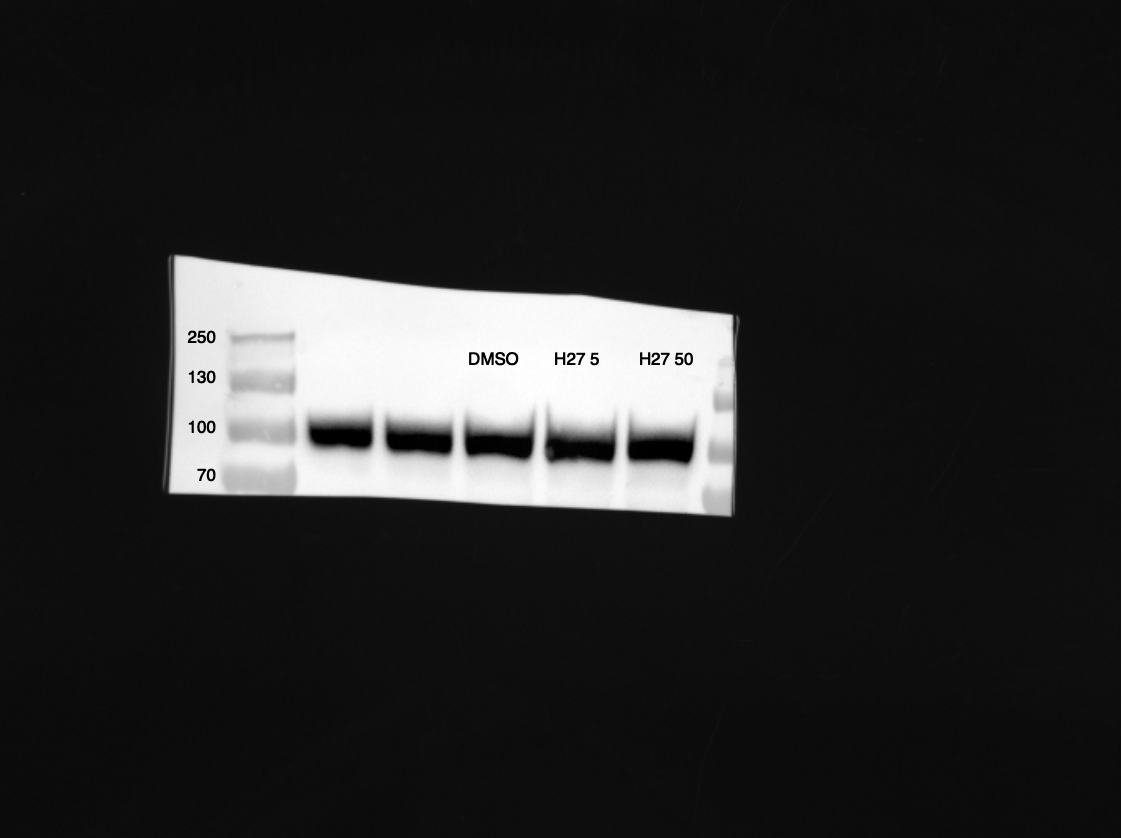

Supplement: Supplementary file 14 — Source data Fig. 5 [file 44321_2024_143_MOESM14_ESM.zip › Figure 5/5B/5B total TRN1 western.tif]

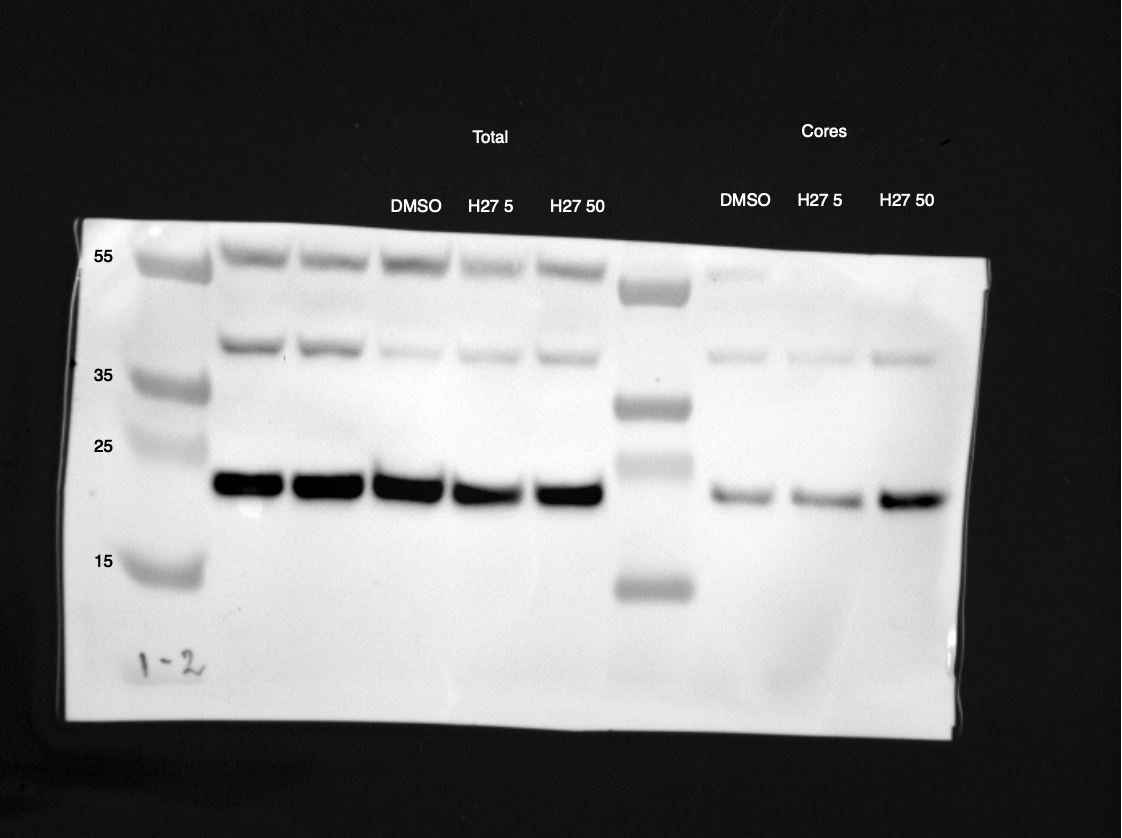

Supplement: Supplementary file 14 — Source data Fig. 5 [file 44321_2024_143_MOESM14_ESM.zip › Figure 5/5B/5B total & cores CA western.tif]

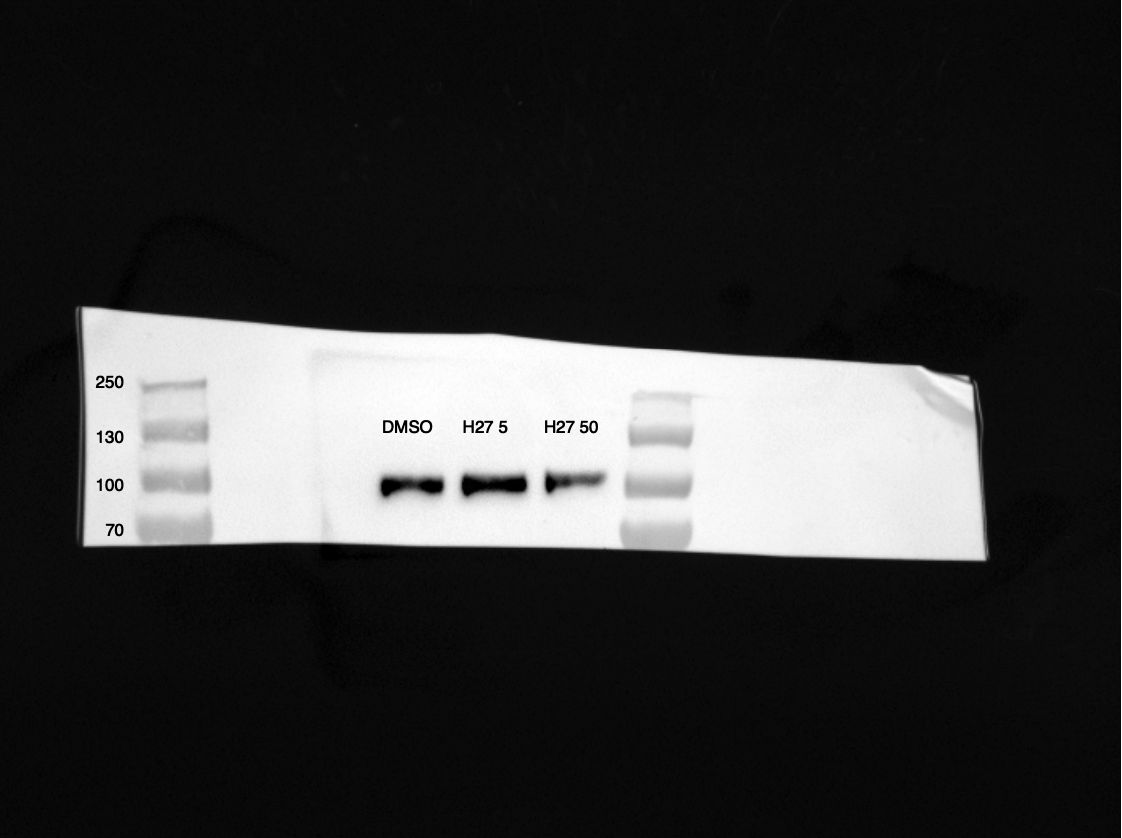

Supplement: Supplementary file 14 — Source data Fig. 5 [file 44321_2024_143_MOESM14_ESM.zip › Figure 5/5B/5B cores TRN1 western.tif]
