# Supplementary material for: A new class of capsid-targeting inhibitors that specifically block HIV-1 nuclear import
Source: EMBO Mol Med. 2024 Oct 2;16(11):13. doi: 10.1038/s44321-024-00143-w (PMC11555092; doi:10.1038/s44321-024-00143-w)
Supplement: Supplementary file 16 — Expanded View Figures [file 44321_2024_143_MOESM16_ESM.pdf]

Expanded View Figures

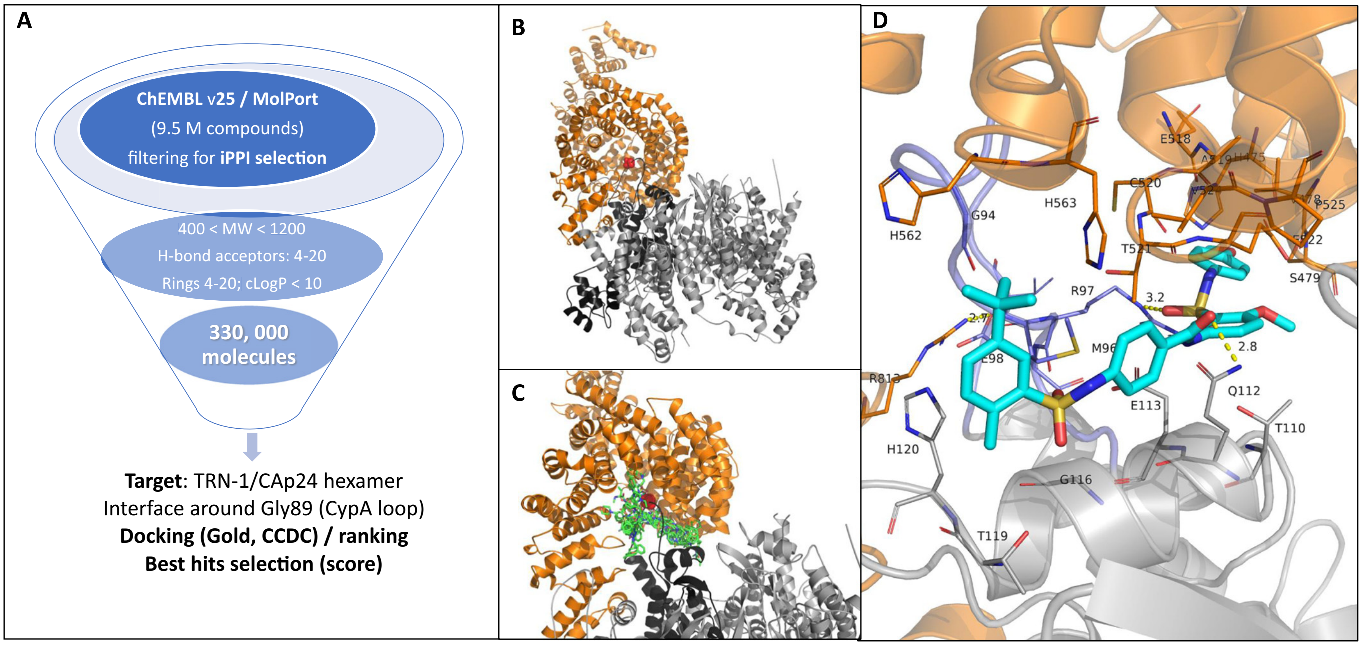

Figure EV1. Workflow for discovering new inhibitors of TRN-1 / CA hexamer interface.

(**A**) Processing of the chEMBL (release 25, 1.9 M molecules) and MolPort (7.6 M of compounds) databases by applying specific criteria to enrich the final library in iPPI (higher molecular weight, lower solubility and high number of hydrogen bond acceptors or rings) resulting in 330,00 final compounds. (**B**) TRN-1/CA hexamer complex obtained by rigid docking. TRN-1 is depicted in orange cartoon representation and CA in grey; Gly89 from the CypA loop is shown as red spheres. (**C**) Illustration of the virtual screening using iPPI compounds with the superimposition of 10 first best-ranking hits obtained by docking with the Gold programme (green sticks). (**D**) Zoom-in view of the interface with residues from TRN-1 or CA interacting with the hit H27 (depicted as cyan sticks).

 © The Author(s)

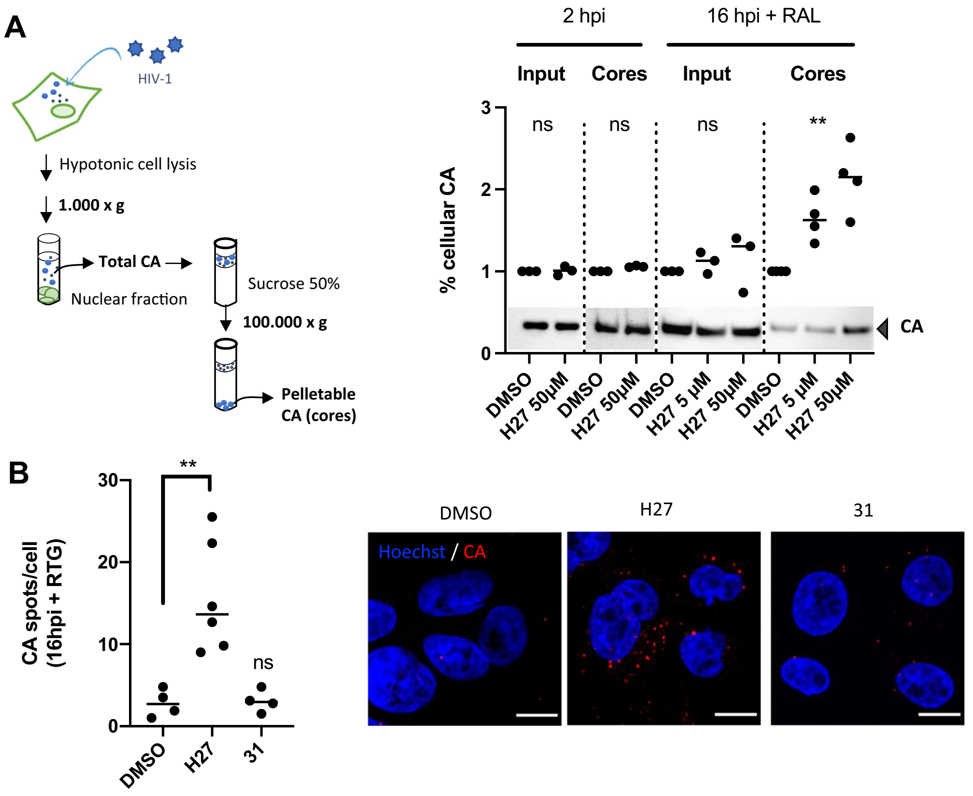

Figure EV2. CA accumulation in cells treated with H27.

(**A**) Fate-of-capsid assay. HeLa-R5 cells were treated with 5 or 50 µM H27 for 2 h, then inoculated with LAI-VSV-G at 1 ng p24/1000 cells for 4 h in serum-free medium to promote cell attachment, and incubated in 10% FCS for a further 2 or 16 h to allow infection to proceed. For the latter time point, cells were treated additionally with RAL (10 µM) to prevent de novo gag synthesis. Cells were collected and lysed in hypotonic buffer, and lysates were either directly analysed by immunoblotting (Input), or were centrifuged through a sucrose cushion to separate pelletable CA (cores) from soluble CA. Results show the immunoblot quantifications from three independent experiments, normalised for the DMSO control. A representative blot is provided below. Statistical significance was assessed by paired non-parametric *t*-test at 2 hpi (Wilcoxon test) and One-way ANOVA at 16 hpi (Friedman test). $^{**}p \leq 0.01$, ns non-significant. (**B**) HeLa-R5 cells were treated with 50 µM H27 or compound **31**, then infected with LAI-VSV-G at 1 ng p24/1000 cells. The effect of compounds on HIV-1 CA accumulation was assessed at 16 hpi with RAL to prevent de novo Gag synthesis. Cells were labelled with anti-CA antibody, stained with Hoechst, and imaged using an LSM880 confocal microscope with 63 x objective. Analysis was performed using the spot detection tool in Imaris 9. The graph indicates the average number of spots per cell for 4 independent experiments and a total of >400 cells. Statistical significance was assessed by ordinary one-way ANOVA with multiple comparison. $^{**}p \leq 0.01$, ns non-significant. Representative confocal images show CA and Hoechst staining. Scale bar = 10 µm.

© The Author(s)

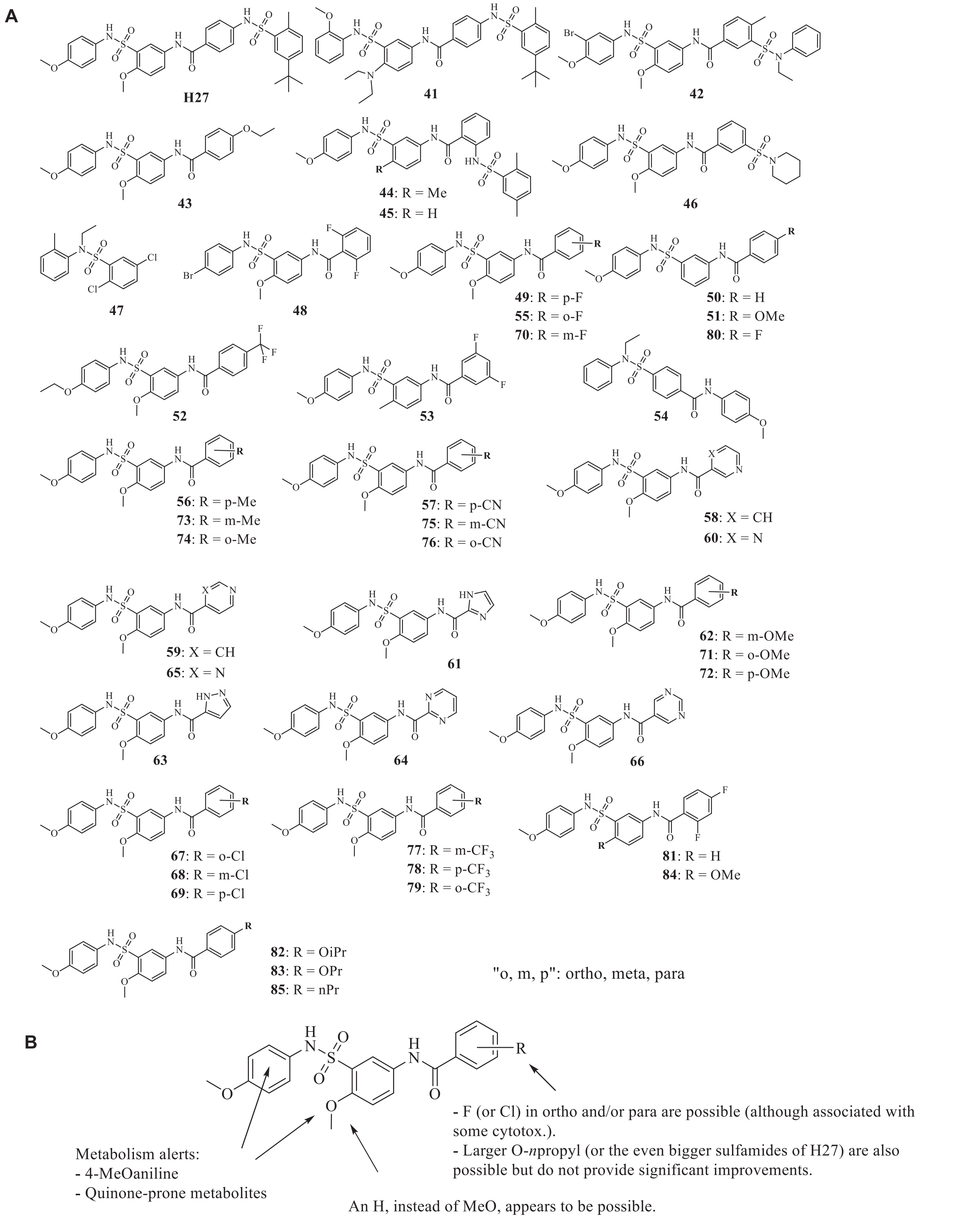

Figure EV3. Structure activity relationship (SAR).

(**A**) Chemical structures of the H27 analogues 41 to 85. (**B**) Conclusions from the structure-activity relationship study.

 © The Author(s)

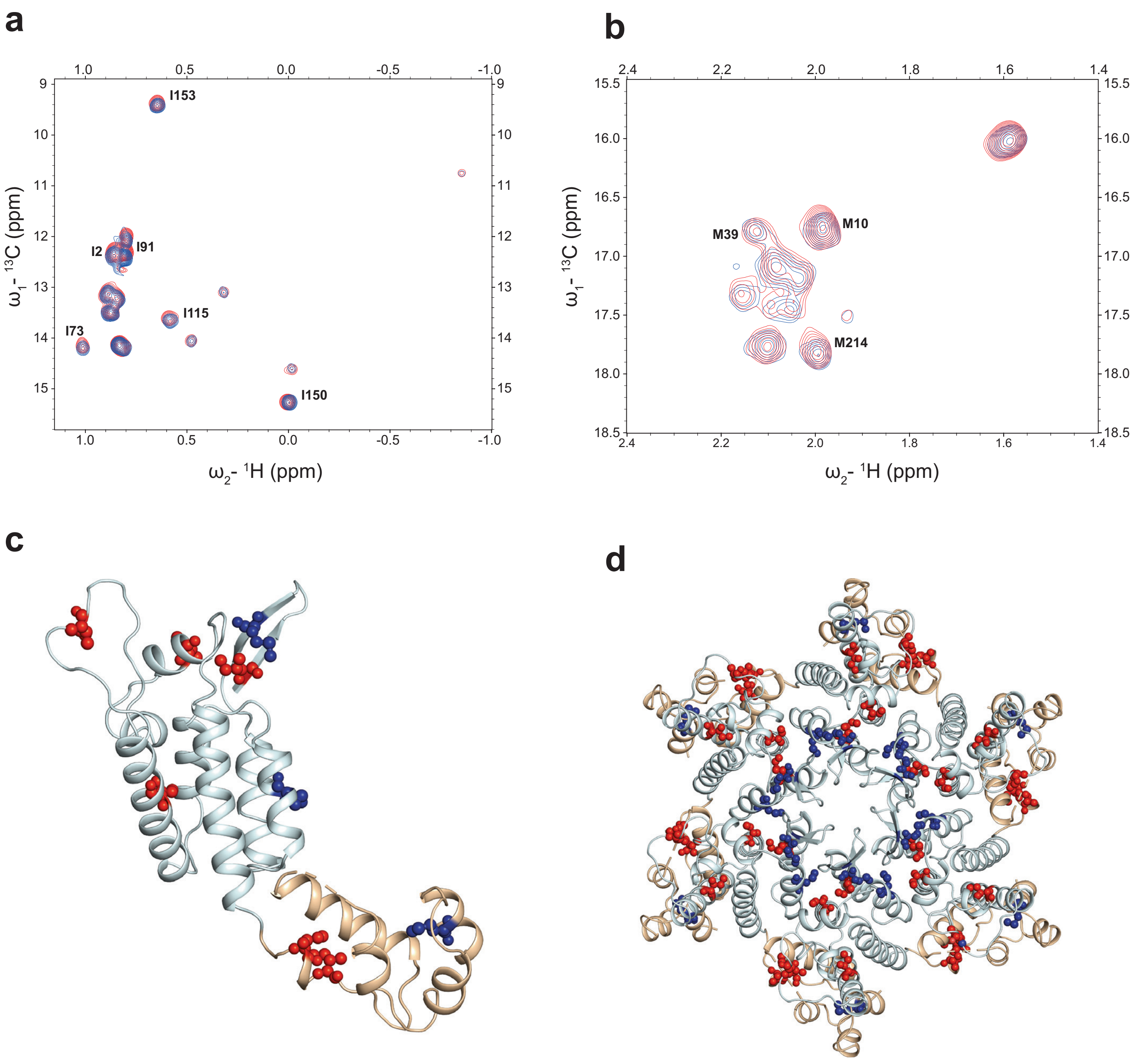

Figure EV4. ^{1}H-^{13}C methyl-TROSY NMR mapping of CA-Hex H27 interactions.

(**A**, **B**) ^{1}H-^{13}C HMQC methyl-TROSY spectra of 50 μM ^{13}C Ile-δ1 - Met-ε labelled CA-hexamer (blue) and upon addition of 1 mM H27 (red). The Ile region of the spectrum is shown in (**A**) and the Met region in (**B**). Introduction of H27 results in only very small chemical shift perturbation <0.1 ppm of the assigned resonances, indicated. (**C**, **D**) Crystal structure of a CA-monomer (**C**) and CA-hexamer (**D**) taken from, PDB ID: 7ZUD. The protein backbone is shown in cartoon representation, CA-NTD is coloured cyan and CA-CTD is coloured wheat. Ile and Met residues displaying chemical shift perturbation are shown in stick and ball representation mapped onto the CA-monomer and hexamer structures, Ile in red and Met in blue. Residues that do show small chemical shift perturbations are largely dispersed across the structure.

© The Author(s)

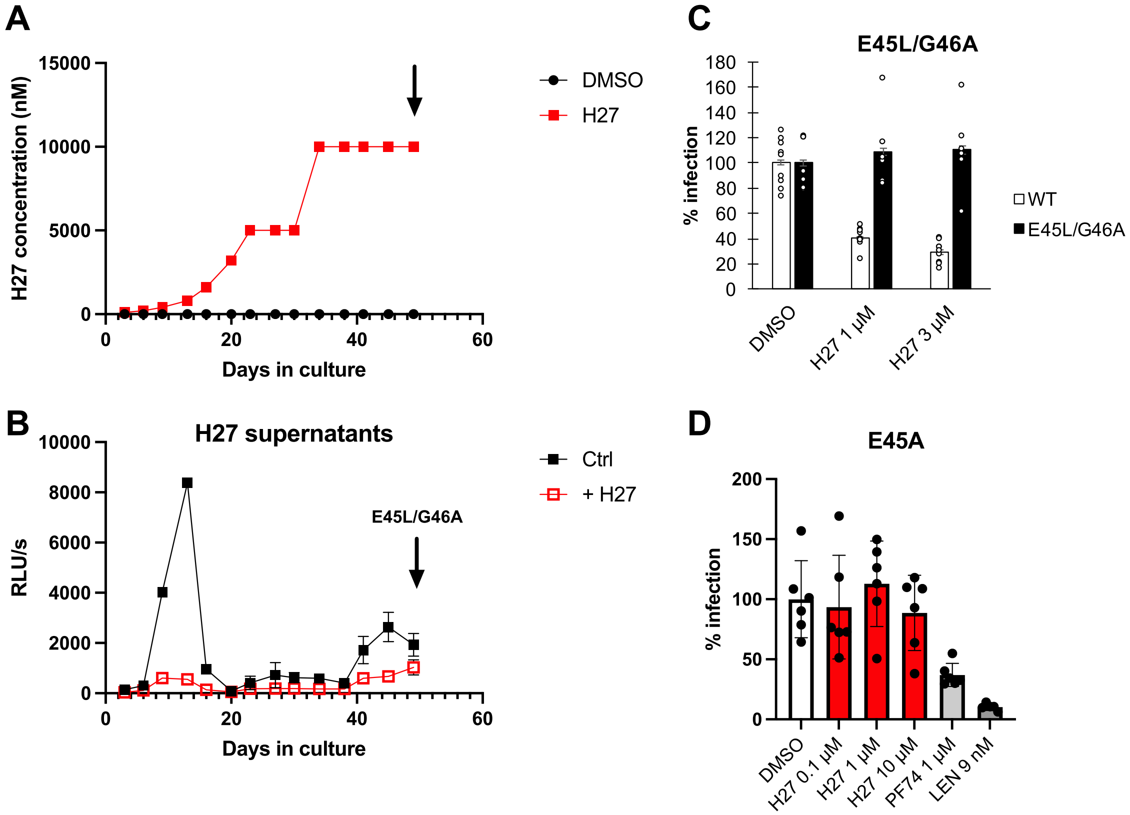

Figure EV5. Capsid mutants that escape H27.

(**A**) JLTR-R5 cells were inoculated with NL4-3 replicative virus at MOI 0.1 in the presence of 100 nM H27. Viral replication was assessed by tat transactivation of the LTR-eGFP reporter and H27 was increased by increments of 2–3-fold starting with 0.2 µM on day 1, until 10 µM from day 34 onwards. Control infections were performed in the presence of DMSO and NVP. (**B**) Virus production and sensitivity to H27 (10 µM) was assessed by inoculating HeLa-R5 cells with 25 µl supernatants from JLTR-R5 cultures, which is ~1:80th of the supernatant volume. β-galactosidase activity indicative of single-cycle infection was assessed at 48 hpi. Results are the mean of 3 independent experiments ± SD. The supernatants at D49 (arrow) exhibited some resistance to H27. TOPO-cloning and sequencing of capsid sequences uncovered a double E45L/G46A escape mutation. (**C**) Site-directed mutagenesis was performed to introduce the E45L/G46A mutations in pNL4-3. Sensitivity to H27 was tested by comparing it with wild-type virus in HeLa-R5 cells. Results show individual and mean values from three independent experiments ± SEM. (**D**) Sensitivity of the E45A mutant to H27. HeLa-R5 cells were treated with 0.1, 1 or 10 µM H27, 1 µM PF74 or 9 nM LEN, then infected with the E45A R9 virus. Results show the mean normalised infectivity values ± SD obtained at 48 hpi from three independent experiments.

 © The Author(s)
